# Three-dimensional super-resolution microscopy of the inactive X chromosome territory reveals a collapse of its active nuclear compartment harboring distinct Xist RNA foci

**DOI:** 10.1186/1756-8935-7-8

**Published:** 2014-04-28

**Authors:** Daniel Smeets, Yolanda Markaki, Volker J Schmid, Felix Kraus, Anna Tattermusch, Andrea Cerase, Michael Sterr, Susanne Fiedler, Justin Demmerle, Jens Popken, Heinrich Leonhardt, Neil Brockdorff, Thomas Cremer, Lothar Schermelleh, Marion Cremer

**Affiliations:** 1 Biocenter, Department of Biology II, Ludwig Maximilians University (LMU), Martinsried, Germany; 2 Department of Biochemistry, University of Oxford, Oxford, UK; 3 Institute of Statistics, Ludwig Maximilians University (LMU), Munich, Germany

**Keywords:** Super-resolution microscopy, X chromosome inactivation, Inactive X chromosome, Chromosome territory, CT, Xist RNA, Barr body, Chromatin domain, Interchromatin compartment, SAF-A

## Abstract

**Background:**

A Xist RNA decorated Barr body is the structural hallmark of the compacted inactive X territory in female mammals. Using super-resolution three-dimensional structured illumination microscopy (3D-SIM) and quantitative image analysis, we compared its ultrastructure with active chromosome territories (CTs) in human and mouse somatic cells, and explored the spatio-temporal process of Barr body formation at onset of inactivation in early differentiating mouse embryonic stem cells (ESCs).

**Results:**

We demonstrate that all CTs are composed of structurally linked chromatin domain clusters (CDCs). In active CTs the periphery of CDCs harbors low-density chromatin enriched with transcriptionally competent markers, called the perichromatin region (PR). The PR borders on a contiguous channel system, the interchromatin compartment (IC), which starts at nuclear pores and pervades CTs. We propose that the PR and macromolecular complexes in IC channels together form the transcriptionally permissive active nuclear compartment (ANC). The Barr body differs from active CTs by a partially collapsed ANC with CDCs coming significantly closer together, although a rudimentary IC channel system connected to nuclear pores is maintained. Distinct Xist RNA foci, closely adjacent to the nuclear matrix scaffold attachment factor-A (SAF-A) localize throughout Xi along the rudimentary ANC. In early differentiating ESCs initial Xist RNA spreading precedes Barr body formation, which occurs concurrent with the subsequent exclusion of RNA polymerase II (RNAP II). Induction of a transgenic autosomal Xist RNA in a male ESC triggers the formation of an ‘autosomal Barr body’ with less compacted chromatin and incomplete RNAP II exclusion.

**Conclusions:**

3D-SIM provides experimental evidence for profound differences between the functional architecture of transcriptionally active CTs and the Barr body. Basic structural features of CT organization such as CDCs and IC channels are however still recognized, arguing against a uniform compaction of the Barr body at the nucleosome level. The localization of distinct Xist RNA foci at boundaries of the rudimentary ANC may be considered as snap-shots of a dynamic interaction with silenced genes. Enrichment of SAF-A within Xi territories and its close spatial association with Xist RNA suggests their cooperative function for structural organization of Xi.

## Background

Sex chromosome dosage differences between male and female mammals are compensated by epigenetic silencing of most genes on one of the two X chromosomes in females to ensure similar transcript levels in both sexes (for reviews see Heard [1], Payer and Lee [2] and Pontier and Gribnau [3]). An early note to understand the route of X chromosome inactivation (XCI) came from Barr and Bertram in 1949 [4], who observed in neuronal cells of cats a small, nucleolus-associated body specific to female nuclei. This so-called Barr body is highlighted from surrounding chromatin by its intense DNA staining and apparent compactness, hinting at a major chromatin condensation. In 1961, Lyon proposed a link between genetic inactivation of one of the two X chromosomes in females and the Barr body, representing the inactive X chromosome (Xi) [5].

X chromosome-specific gene silencing starts at early embryogenesis [5-7] (for review see Payer *et al*. [8]). Initiation of XCI requires the expression and spreading of the non-coding X inactive specific transcript (Xist) RNA *in cis* along the later Xi [9] (reviewed in Pontier and Gribnau [3] and Brockdorff [10]). Xist RNA spreading is followed by a gradual loss of active chromatin marks such as trimethylated histone H3 lysine 4 (H3K4me3) and enrichment of repressive marks, for example trimethylated histone H3 lysine 27 (H3K27me3), incorporation of the histone variant macroH2A1, and finally DNA methylation, together mediating the chromosome-wide silencing of gene activity [11] (reviewed in Heard *et al*. [12], Jeon *et al*. [13] and Yang *et al*. [14]). Xist RNA was shown to be involved in mediating the particular chromosome conformation seen as the Barr body [15,16]. However, it is not known at what time point during the XCI process chromatin compaction towards a Barr body occurs.

Chromosomes occupy distinct territories (chromosome territories; CTs) in the interphase nucleus [17]. Increasing experimental evidence supports a functional organization of CTs composed of a chromatin compartment (CC) represented by interconnected, approximately 1 Mb sized chromatin domain clusters (CDCs) [17-19] and an interchromatin compartment (IC). The CC and IC form two spatially contiguous and functionally interacting networks throughout the nuclear space [20-25]. Transmission electron microscopic (TEM) studies have provided evidence that compacted CDCs are lined by a perichromatin region (PR), a layer of approximately 100 nm of decondensed chromatin, which constitutes the interface between IC and CC (reviewed in Fakan and van Driel [26]). The PR was found to be enriched both in nascent RNA and nascent DNA [27,28], and was thus suggested as the nuclear subcompartment for transcription and DNA replication. The IC was defined as a nearly chromatin-free channel system starting at nuclear pores and pervading between the higher-order CDC network that serves as a system for the allocation of components needed within the PR, as well as for the guided diffusion of macromolecules [29-31].

Previous observations based on conventional fluorescence microscopy have described a compacted sphere-like Xi/Barr body in contrast to a flat and extended active X (Xa) territory [32-34]. This strongly suggests a major difference in higher-order chromatin organization between Xa and Xi territories. Earlier studies addressing the subchromosomal structure of the Barr body found X chromosomal genes preferentially located in a concentric layer around the compacted, Xist RNA decorated Barr body, either correlated [15,35] or independent [32] of their transcriptional activity. More recently, both genes silenced by XCI as well as escapees were found throughout the entire Barr body [34,36]. Furthermore, a non-uniform compaction behavior of subchromosomal segments in relation to the addressed genomic distance was noted: higher compaction in Xi territories was found for chromosomal segments of approximately 20 Mb, but was not accordingly reflected in enclosed segments of approximately 1 Mb [34]. This non-uniformity of chromatin compaction hinted at local compaction differences within Barr bodies as a consequence of a differential reorganization of higher-order structures and argued against a uniformly increased compaction at the nucleosome level. In three-dimensional (3D) reconstructions from ultrathin TEM serial sections in the Barr body of human and mouse fibroblast nuclei, tightly packed chromatin fibers separated by interchromatin tunnels with direct connections to nuclear pores were described [37].

To date, we still lack comprehensive information on the basic principles and fundamental differences in organization of Xi and transcriptionally competent CTs, the process of X chromosomal compaction, and the spatial arrangement of Xist RNA in relation to particular features of the Xi at the single-cell level. In part, this lack of structural knowledge has been a consequence of technical limitations, most prominently the diffraction limited optical resolution of conventional fluorescence microscopy and the inherent difficulties of electron microscopy (EM) to explore the 3D topography of multiple structural components. Recent super-resolution microscopy techniques have made it possible to overcome these limitations (for reviews see Cremer *et al*. [38], Hell [39], Huang *et al*. [40], Rouquette *et al*. [41] and Schermelleh *et al*. [42]). Of these approaches, 3D structured illumination microscopy (3D-SIM), allows sub-diffraction multicolor far-field optical sectioning with a two-fold resolution improvement in each spatial dimension resulting in an approximate eight-fold increased volumetric resolution [43]. This makes this technique particularly suited for the 3D analysis of nuclear ultrastructures and their spatial relationships [29,31,44,45].

In this study we employed 3D-SIM to explore the 3D organization of the Barr body in human and mouse somatic cells compared to transcriptionally competent CTs and to validate currently discussed models of the Xi ultrastructure (reviewed in Arthold *et al*. [46] and Wutz [47]). We provide evidence that the Barr body is composed of compacted CDCs and an IC/PR network and shares these principal structural features with all other CTs. Since small chromatin loops may expand to some extent into the IC [29,41,48] we defined the IC/PR here as a complex and functionally coherent compartment, and termed it the active nuclear compartment (ANC). We find that the Barr body is characterized by a partially collapsed ANC that in contrast to the expanded ANC of transcriptionally competent CTs lacks RNA polymerase II (RNAP II) and H3K4me3. We observed distinct Xist RNA foci localized in close association with the nuclear matrix protein scaffold attachment factor-A (SAF-A) both within and at the boundary zone of the collapsed ANC permeating the entire Barr body. Accordingly, we found little colocalization between Xist RNA foci and H3K27me3-enriched chromatin marking the compact CDCs of the Xi territory. In early differentiating female mouse embryonic stem cells (XX ESCs) we observed initial spreading of Xist RNA as distinct foci before chromatin compaction. Formation of a Barr body congruent with the ‘Xist RNA territory’ was observed with subsequent (gradual) RNAP II exclusion. In a male ESC line with an autosomal inducible Xist transgene (described in Wutz and Jaenisch [49]), we found Xist RNA foci persistently extending into decondensed and apparently active chromatin regions. This finding underlines the importance of the X chromosomal chromatin context for proper Xist RNA propagation and effective transcriptional repression. Our observations highlight general principles of higher-order chromatin organization in mammalian genomes. In light of the recent observation of Xist binding broadly across the Xi obtained by an RNA antisense purification method [50], our single-cell observations of a focal representation of Xist RNA suggest their dynamic association at different sites of the collapsed ANC.

## Results

### A re-evaluation of chromatin organization in the Barr body by 3D-SIM

**Figure 1:**
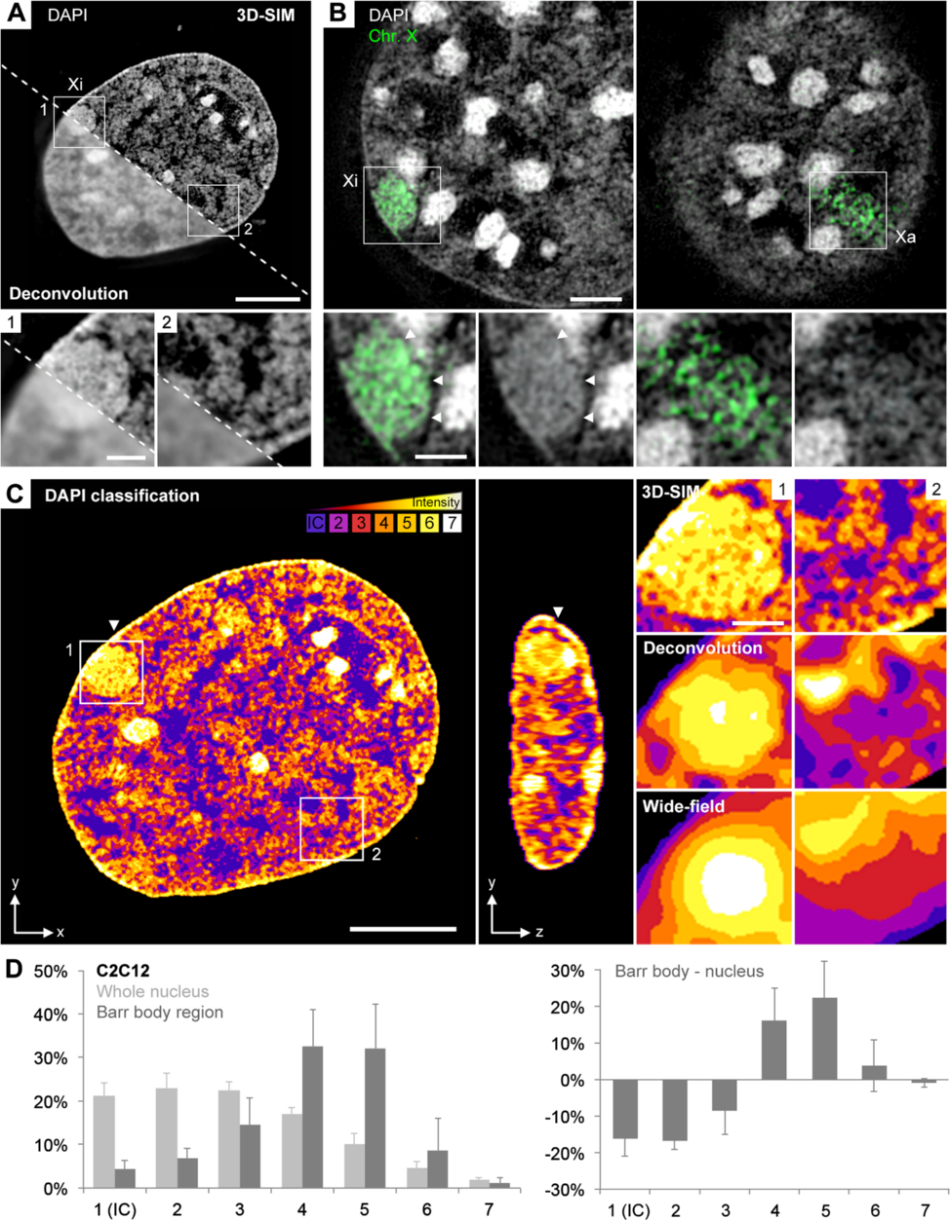
**3D-SIM-based DAPI intensity classification in the Barr body versus the entire nucleus of C2C12 cells. (A)** Mid z-section of a DAPI-stained nucleus. The area below the dashed line illustrates the resolution level obtained by wide-field deconvolution microscopy, for comparison*.* Inset magnifications show the non-uniformly compacted structure of the Barr body resolvable with 3D-SIM (1) and an arbitrary autosomal region with CDCs (2). Scale bars: 5 μm, insets 1 μm. **(B)** X chromosome-specific painting (green) of Xi (left) and Xa territories (right) of the same nucleus in different z-sections. Note the high convergence between the painted Xi and the DAPI visualized Barr body (arrowheads). Scale bars: 2 μm, insets 1 μm. **(C)** 3D DAPI intensity classification exemplified for the nucleus shown in **(A)**. Seven DAPI intensity classes displayed in false-color code ranging from class 1 (blue) representing pixels close to background intensity, largely representing the IC, up to class 7 (white) representing pixels with highest density, mainly associated with chromocenters. Framed areas of the Barr body (inset 1) and a representative autosomal region (inset 2) are shown on the right at resolution levels of 3D-SIM, deconvolution and conventional wide-field microscopy. The Xi territory pervaded by lower DAPI intensities becomes evident only at 3D-SIM resolution, whereas both wide-field and deconvolution microscopy imply a concentric increase of density in the Barr body. In the autosomal region, chromatin assigned to classes 2 to 3 lines compacted CDCs, represented by classes 4 to 6. **(D)** Left: average DAPI intensity classification profiles with standard deviations evaluated for entire nuclear volumes or the Barr body region only (dark grey bars). Right: over/underrepresentation of the average DAPI intensity class fraction sizes in the Barr body versus entire nuclear volumes (n = 12). Distribution differences on classes between Xi and entire nucleus *P* <0.001. 3D-SIM, three-dimensional structured illumination microscopy; CDC, chromatin domain cluster; DAPI, 4',6-diamidino-2-phenylindole; FISH, fluorescence *in situ* hybridization; IC, interchromatin compartment; Xa, active X chromosome; Xi, inactive X chromosome.

At the resolution level of 3D-SIM we compared the subchromosomal organization of the Barr body constituting a transcriptionally repressed CT with transcriptionally competent chromatin represented by autosomes and the Xa in female mouse C2C12 (Figure 1) and human RPE-1 cells (Additional file 1). In both cell types, the Barr body was highlighted by its intense DAPI staining and clearly demarcated from surrounding, less condensed chromatin. Moreover, a distinct folding substructure of DAPI-stained chromatin in the Barr body became evident with 3D-SIM, which could hardly be resolved by diffraction-limited wide-field microscopy even after deconvolution (Figure 1A, left panel). DNA-fluorescence *in situ* hybridization (FISH) painted Xi territories largely coincided with DAPI-delineated Barr bodies indicating that the Barr body comprises the entire Xi territory in these cell types (Figure 1B). Accordingly, painted X chromosome territories would allow a direct comparison between the 3D structure of the Barr body (Xi) and its Xa counterpart. 3D-FISH, however, typically requires heat denaturation of DNA, which might impede ultrastructural details of chromatin and proteins [44,51]. Therefore, we avoided DNA denaturation and verified the Barr body/Xi by Xist RNA-FISH. Its chromatin landscape was compared against the entire nucleus reflecting the landscape of autosomal CTs and the active X.

Using a novel tailored 3D segmentation algorithm, DAPI-stained DNA signals were divided into seven intensity classes with equal intensity variance (Figure 1C). This classification was a deliberate simplification (compared to, for example, 65,536 grey levels in 16-bit images) but provided a clear visualization of nuclear landscapes shaped by different DAPI intensities and allowed for a statistical comparison between different nuclear areas or samples. Class 1 represented regions close to background intensities, suggesting a largely DNA-free compartment. Classes 2 and 3 represented chromatin of low staining intensity, which lined the more compacted CDCs represented by classes 4 to 6. Class 7 represented the highest DAPI intensities and mostly delineated the chromocenters in C2C12 nuclei. The respective DAPI classification in the Barr body also revealed a chromatin network of lower intensities pervading throughout the Xi territory (Figure 1C, inset 1). Note that in contrast to Barr body classifications carried out on SIM images, classifications based on wide-field microscopy images before and after deconvolution suggested highest DNA densities in the center of the Barr body gradually decreasing towards its periphery (inset magnifications in Figure 1C). This exemplifies an erroneous interpretation due to limits of microscopic resolution. DAPI intensity classifications of both entire nuclear volumes and of Barr body regions only confirmed the representation of all classes in the Barr body in both cell lines (Figure 1D). Compared with autosomal regions, low intensities, in particular classes 1 and 2 were, however, significantly underrepresented.

The suitability of DAPI as a marker for global chromatin representation despite its reported binding preference to AT-rich DNA [52] was verified by control stainings with SYTOX Green, a nucleic acid stain without sequence preference. This resulted in a similar classification profile of DNA intensities which was also seen with H2B-GFP-tagged nuclei (Additional file 2, see this file also for the applicability of SYTOX with different 3D-SIM microscope setups). The X chromosome is above average in its AT content [53], thus chromatin density classification for the Barr body based on DAPI intensities could be biased. These concerns were addressed by assessing DAPI intensity profiles of both Xi and Xa territories after 3D-FISH using X chromosome-specific painting probes (for a detailed explanation see Additional file 3).

**Figure 2:**
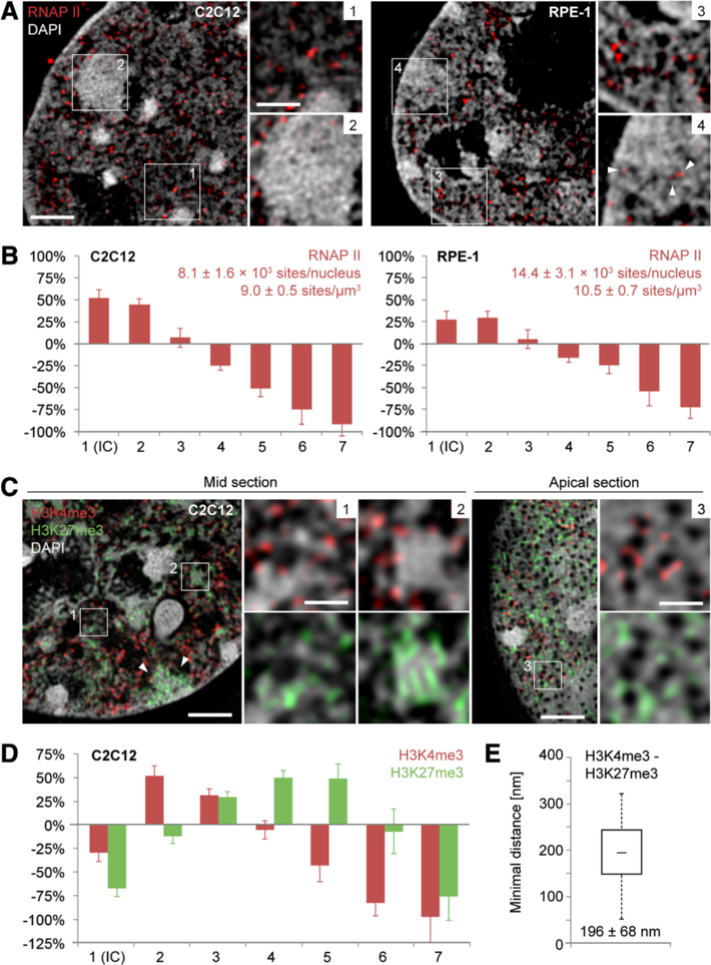
**Topological chromatin density mapping of functionally relevant markers RNAP II, H3K4me3 and H3K27me3. (A)** Mid z-sections through a C2C12 and an RPE-1 nucleus show abundant RNAP II foci preferentially at the boundary of chromatin and IC (insets 1 and 3). RNAP II signals are largely excluded from the Barr body in C2C12 (inset 2), while RPE-1 cells retain some sites of active transcription in the Barr body interior (inset 4, arrowheads; Barr bodies verified by Xist RNA, not shown here). Scale bars: 2 μm, insets 1 μm. **(B)** Over/underrepresentation of RNAP II in DAPI intensity classes of C2C12 (n = 7) and RPE-1 (n = 7) nuclei relative to the intensity class sizes as shown in Figure 1D and Additional file 1. Average RNAP II foci numbers and densities are indicated with standard deviations (*P* <0.001). **(C)** Clear separation of H3K4me3- and H3K27me3-marked chromatin shown in a mid (left) and apical z-section (right) of a C2C12 nucleus (arrow delineates the Barr body). H3K4me3 is located mainly at the decondensed periphery of CDCs, whereas H3K27me3 is enriched within compacted CDCs (insets 1 and 2). In the apical z-section H3K4me3-enriched chromatin is largely restricted to the vicinity of nuclear pores, whereas H3K27me3 is also found at more distant areas. Scale bars: 2 μm, insets 0.5 μm. **(D)** Comparative mapping of H3K27me3 (green) and H3K4me3 (red) signals on DAPI intensity classes in C2C12 nuclei (n = 10, distribution differences on classes *P* <0.001 for all markers). **(E)** Minimal distance distributions (nearest neighbor distances) for H3K27me3 and H3K4me3 signals displayed as box plots (median, Q1, Q3) with whiskers indicating the 1.5 IQR. Average minimal distances indicated with standard deviation (>100,000 distances determined from 20 cells; see Additional file 4 for all minimal distance distributions determined in this study). 1.5 IQR, 1.5 × interquartile range; CDC, chromatin domain cluster; DAPI, 4',6-diamidino-2-phenylindole; H3K27me3, trimethylated histone H3 lysine 27; H3K4me3, trimethylated histone H3 lysine 4; IC, interchromatin compartment; RNAP II, RNA polymerase II.

We further substantiated the functional link between the topological chromatin density landscape and its biological relevance by quantitatively mapping the relative spatial distribution of immunodetected RNAP II, H3K4me3 and H3K27me3, markers for transcriptionally competent and repressed chromatin, respectively [54] on the seven DAPI intensity classes (Figure 2A,B,C,D). This approach was complemented by the measurement of minimal distances (nearest neighbor analysis) between the differently labeled fluorescent signals (Figure 2E). The average number and density of RNAP II sites (approximately 8,000 per nucleus in C2C12 cells and 14,000 in RPE-1 cells; approximately 9 sites/μm^3^ and 10 sites/μm^3^, respectively) were in the same range as estimated for HeLa and other cells from light and EM imaging of cryosections [55]. In line with their functional assignment, signals reflecting actively elongating RNAP II were distinctly overrepresented in the two lowest DAPI intensity classes 1 and 2 (Figure 2B). Notably, RNAP II sites were almost fully excluded from Barr bodies in C2C12 cells, while RPE-1 cells consistently retained a few RNAP II sites even in the interior of Barr bodies (Figure 2A), likely reflecting the higher fraction of escapees in Xi of human compared to mouse (15% versus 3%) [56].

Both in the Barr body and throughout the nucleus, H3K4me3 was found enriched at decondensed sites at the boundary of CDCs and IC channels, while H3K27me3 labeling sites were preferentially, although not exclusively, located in the more compacted interior of CDCs (Figure 2C). At the nuclear periphery, representing a transcriptionally largely but not completely repressed nuclear compartment [57], H3K4me3 was found closely associated with nuclear pores, recently defined as potential sites of transcriptional activity [58] (Figure 2C, right). H3K4me3 mapping to DAPI intensity classes revealed the most pronounced overrepresentation in the low intensity classes 2 and 3, while H3K27me3 overrepresentation was most evident in the intermediate classes 4 to 5 (Figure 2D). Notably, in contrast to RNAP II, H3K4me3 was underrepresented in class 1 (comprising the largely DNA-free IC), and both H3K4me3 and H3K27me3 were distinctly underrepresented in class 7 (chromocenters), illustrating their role as gene silencing/activation marks. The average minimal distance of around 200 nm between H3K4me3- and H3K27me3-marked chromatin signals in a nearest neighbor analysis confirmed their spatial separation (Figure 2E; see Additional file 4 for a comparative overview of all minimal distance measurements of this study).

The distinct nuclear landscapes shaped by different (DAPI-defined) chromatin density classes, their correlation with functionally distinct biological markers, together with previous experimental evidence as outlined in the introduction, justified the assignment of low intensity classes to the ANC comprising the functionally coherent IC and PR. Accordingly, we considered the underrepresentation of classes 1 to 3 in the Barr body, which exemplifies a globally transcriptionally repressed CT, as the result of a narrowing or even partial collapse of the ANC in the Xi.

### Barr body structure maintains a contiguous, dense 3D chromatin network with a collapsed ANC channel system

**Figure 3:**
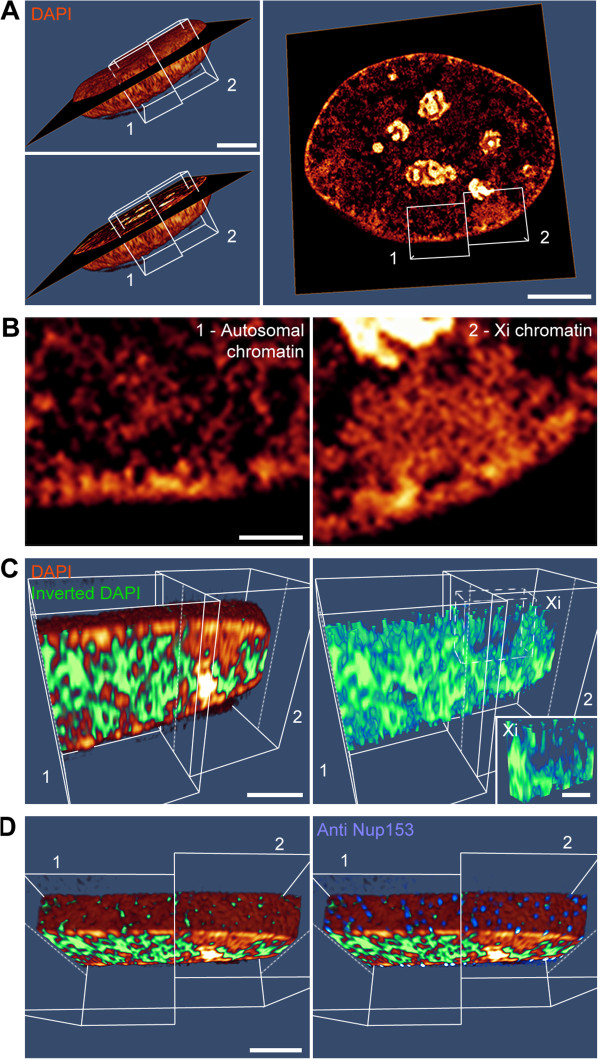
**3D visualization of an interconnected ANC channel network leading to nuclear pores in autosomal and Barr body regions. (A)** 3D volume rendering of a DAPI-stained C2C12 nucleus imaged with 3D-SIM. Entire nuclear volume (left panels) and mid z-section (right panel) is shown. White boxes mark the regions shown in detail in **(B,C,D)**. Scale bar: 5 μm. **(B)** Single z-section from autosomal (left) and Barr body region (right). Note that the autosomal chromatin is pervaded by an ANC network, occasionally forming large IC lacunae. This channel network is distinctly narrowed in the Barr body and lacks larger IC lacunae. Scale bar: 1 μm. **(C)** Left panel: 3D volume rendering of DAPI-stained chromatin (brown) and representation of the inverted DAPI signal within the nuclear interior marking the ANC compartment (green) of the two cuboids depicted in **(A)**. Right panel: inverted DAPI signal only. Inset magnification shows the cropped Barr body with a rudimentary channel system representing the collapsed ANC. Scale bars: 1 μm, inset 0.5 μm. **(D)** Top view of the same region as in **(C)** with green channels leading to the nucleus’ surface in autosomal as well as in Barr body chromatin (left). Nuclear pore complex immunostaining with antibodies against Nup153 (blue) demonstrates the overlap of ANC channel signals (green) at the nuclear surface with nuclear pore complexes (right) (see also respective movies provided in Additional files 5, 6 and 7). Scale bar: 1 μm. 3D-SIM, three-dimensional structured illumination microscopy; ANC, active nuclear compartment; DAPI, 4',6-diamidino-2-phenylindole; IC, interchromatin compartment.

We further aimed to validate whether the Barr body maintains a contiguous 3D ANC channel system, pervading the 3D chromatin network and leading to the nuclear pores (Figure 3). Optical sections of a DAPI-stained C2C12 nucleus denoting the position of the Barr body and a neighboring autosomal region showed irregularly shaped higher-density chromatin clusters pervaded by less intensely or unstained regions. The latter were remarkably wider in nuclear regions representing transcriptionally competent CTs, but also visible in the Barr body (Figure 3A,B) in line with DAPI intensity classifications. A higher-order 3D network of condensed CDCs and a low-density 3D channel system became obvious by 3D volume rendering (Figure 3C) and more evident by following-up these channels in respective movies (Additional files 5, 6 and 7). While nuclear areas harboring active CTs revealed wide-spaced channels and lacunae, the Barr body apparently retained only a rudimentary channel system representing the collapsed ANC. Yet, these channels could be followed from the Barr body interior through peripheral heterochromatin leading to Nup153-stained nuclear pores similar to nuclear regions with active CTs (Figure 3D and Additional file 7), further strengthening the maintenance of basic principles of CT organization in the Barr body.

To judge the potential impact of fixation artifacts on large-scale chromatin organization we performed 3D-SIM live-cell experiments with HeLa cells stably expressing histone H2B-GFP, a commonly used marker for the visualization of chromatin (Additional file 8). Live and fixed cells showed a high degree of similarity with regard to chromatin clusters, sites of decondensed chromatin, IC lacunae and ANC channels leading to nuclear pores. These observations largely ruled out that these features of higher-order chromatin organization, as seen at the level of 3D-SIM resolution, represent fixation artifacts.

### Xist RNA foci are enriched at the boundaries of the collapsed ANC

**Figure 4:**
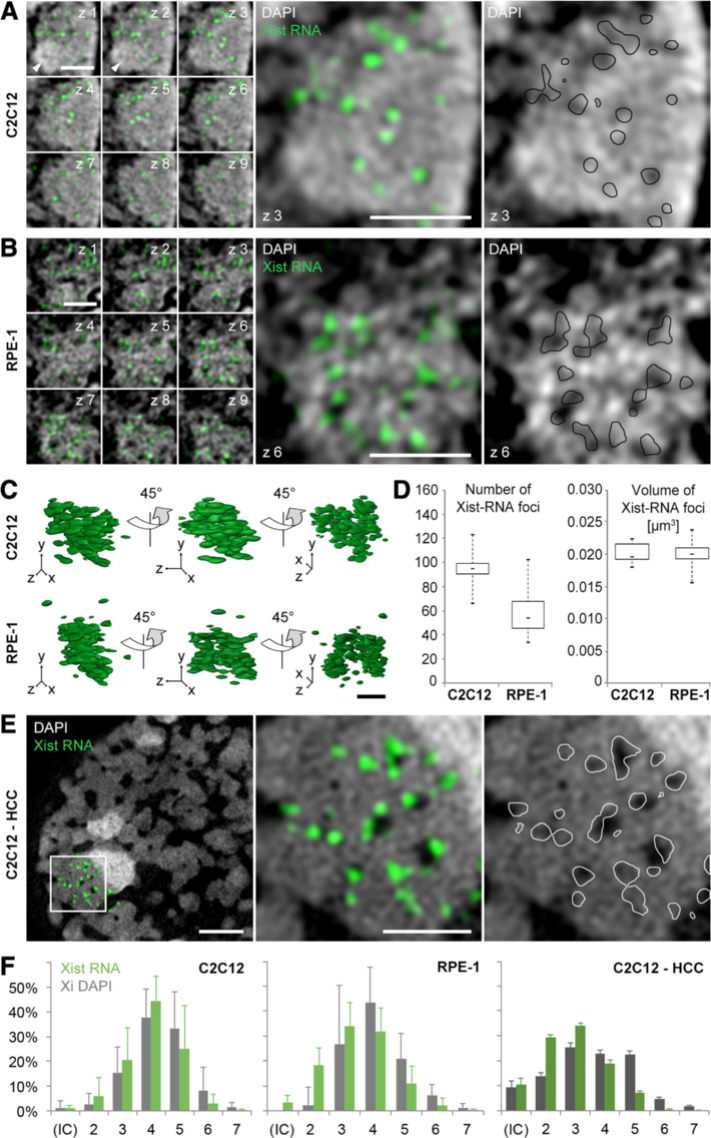
**Xist RNA topography within the Barr body of somatic cells.** Gallery of consecutive 3D-SIM z-sections (125 nm z-distance) through the Barr body of **(A)** a C2C12 and **(B)** a RPE-1 nucleus stained with DAPI (grey) after Xist RNA-FISH (green). Xist RNA penetrates the entire Barr body (with exception of the chromocenter region of C2C12 cells; arrowheads in z 1 and z 2). Scale bars: 1 μm. Higher magnifications (z 3 and z 6, respectively) illustrate the preferential albeit not exclusive localization of Xist RNA along lower intensity DAPI regions. **(C)** 3D surface renderings of Xist RNA foci of the entire Barr bodies shown in **(A)** and **(B)**. **(D)** Boxplots with number and volume distribution of 3D-SIM discernible Xist RNA foci in single Barr bodies of C2C12 (n = 10) and RPE-1 (n = 22) nuclei. Median numbers determined for C2C12 and RPE-1 cells were 95 and 54, median volumes 0.0195 and 0.0198 μm^3^, respectively. **(E)** C2C12 nucleus after induced HCC, resulting in similar chromatin density between the Barr body and surrounding chromatin. Note widening of IC channels in the Xist RNA decorated Barr body and accumulation of Xist RNA foci at their border. Scale bars: 2 μm, inset 1 μm. **(F)** Relative fraction (representation) of Xist RNA signals (green) in Barr bodies of C2C12 (n = 9), RPE-1 (n = 13) and HCC-induced C2C12 cells (n = 14) mapped to each DAPI intensity class (grey) reveal a shift of Xist signals toward lower intensity classes, most prominent after HCC treatment. Distribution differences of Xist on classes *P* <0.001 for all cell types. 3D-SIM, three-dimensional structured illumination microscopy; DAPI, 4',6-diamidino-2-phenylindole; FISH, fluorescence *in situ* hybridization; HCC, hypercondensed chromatin; IC, interchromatin compartment; Xist, X inactive specific transcript.

We next explored in detail the topography of Xist RNA within the Barr body architecture of C2C12 and RPE-1 nuclei by 3D-SIM (Figure 4). Full-length Xist RNA is a 15 kb (mouse) to 17 kb (human) long, non-coding transcript (for reviews see Pontier and Gribnau [3] and Sengupta *et al*. [59]) with an estimated half-life of several hours, relying on a constant turnover of the transcript within the Xi territory [60,61]. By 3D-SIM we identified Xist transcripts as distinct focal structures scattered throughout the Barr body (Figure 4A,B,C). Since our studies were done on fixed cells, these distributions may reflect snap-shots of dynamic positional changes of Xist foci possibly ongoing in living cells. Barr bodies of C2C12 nuclei harbored almost twice the amount of 3D-SIM discernible Xist RNA foci compared to RPE-1 nuclei (medians 95 and 54, respectively), while volumes of individual foci (medians 0.0195 μm^3^ and 0.0198 μm^3^) were almost identical in both cell types (Figure 4D). Xist RNA foci were preferentially found at low to intermediate intensity DAPI-stained chromatin sites, considered as a representation of the collapsed ANC (Figure 4A,B and Additional file 9). Further evidence for Xist RNA localization within this compartment was obtained in experiments that induce a re-opening of collapsed IC channels. For this purpose, we incubated living cells in hyperosmolar medium, which triggers a rapid hypercondensation of chromatin (HCC) concomitant with the widening of preformed IC channels [20,62]. This effect is fully reversible when cells are exposed again to normotonic medium [20]. As expected, widening of the IC could be triggered also within the Barr body of HCC-treated nuclei. Xist RNA foci were observed in these Barr bodies nestling along the borders between compacted CDCs and the widened IC (Figure 4E). Notably, Xist RNA did not completely fill the widened IC suggesting its adhesion to chromatin bordering IC channels. The preferential localization of Xist RNA at lower intensity chromatin sites was confirmed by a quantitative mapping of Xist RNA signals to DAPI intensity classes, which demonstrated their overrepresentation in lower intensity classes both within normotonic and more pronounced within ‘HCC’ Barr bodies (Figure 4F).

### Xist RNA and SAF-A partially overlap in chain-like structures

**Figure 5:**
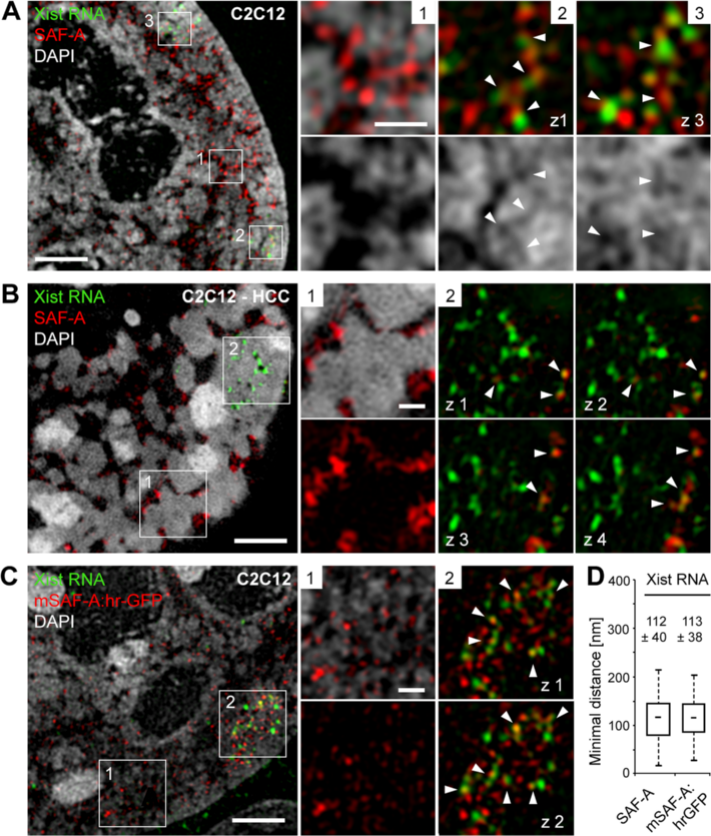
**Spatial association of Xist RNA with the nuclear matrix protein SAF-A.** Immuno-RNA-FISH detection of Xist RNA (green) and SAF-A (red) in C2C12 cells, counterstained with DAPI (grey). **(A)** Immunolabeled endogenous SAF-A is abundant in the IC and at low DAPI intensity sites of random autosomal nuclear regions (inset 1), but scarce within the Xi (insets 2 and 3, representing different z-sections of the nucleus). SAF-A and Xist RNA foci are in close spatial proximity within the narrowed ANC of the Xi (arrowheads). **(B)** C2C12 nucleus with HCC. SAF-A localizes within the widened IC channels (inset 1) and is largely restricted to the periphery of the Xi (inset 2; four consecutive z-sections with step size 0.125 μm). Arrowheads point to Xist RNA foci closely associated to SAF-A signals. **(C)** C2C12 nucleus with transiently expressed hrGFP-tagged murine isoform of SAF-A detected with an antibody against hrGFP (red) together with Xist RNA (green). In comparison to autosomal regions (inset 1) SAF-A:hrGFP is enriched within the Barr body and shows a high degree of association to Xist RNA foci (inset 2). Scale bars **(A,B,C)**: 2 μm, insets 0.5 μm. **(D)** Nearest neighbor distances (minimal distance distribution) with a median of approximately 120 nm for segmented red and green Xist RNA signals to SAF-A (n = 11 nuclei; >400 distances) and mSAF-A:hrGFP (n = 11 nuclei; 270 distances) are displayed as box plots (median, Q1, Q3) with whiskers indicating the 1.5 IQR. Mean values with standard deviations are indicated. 1.5 IQR, 1.5 × interquartile range; ANC, active nuclear compartment; DAPI, 4',6-diamidino-2-phenylindole; FISH, fluorescence *in situ* hybridization; GFP, green fluorescent protein; HCC, hypercondensed chromatin; IC, interchromatin compartment; SAF-A, scaffold attachment factor-A; Xi, inactive X chromosome; Xist, X inactive specific transcript.

Previous studies hinted at a functional interaction between Xist RNA and the nuclear matrix protein SAF-A [63]. This prompted us to analyze the 3D nuclear topography of SAF-A in relation to Xist RNA in the Barr body of C2C12 cells by 3D-SIM (Figure 5). We found immunolabeled SAF-A signals abundantly distributed throughout the nucleus mostly localized at low DAPI intensity sites comprising the ANC (Figure 5A, inset 1). This localization became more obvious after induction of HCC (Figure 5B, inset 1). In the Barr body, immunodetected SAF-A signals were scarce. Yet, the few SAF-A foci typically showed a close spatial proximity or partial overlap with Xist RNA foci, occasionally forming chain-like structures that could weave through the narrow ANC of the Barr body (Figure 5A, insets 2 and 3). In Barr bodies of HCC-treated cells SAF-A was largely absent after immunodetection (Figure 5B, inset 2).

The apparent underrepresentation of SAF-A signals in the Barr body may be due to a Xi-specific conformational switch or post-translational modification on SAF-A leading to epitope masking or hindrance of SAF-A antibody binding and thus inadequate detection of SAF-A epitopes (discussed by Nakagawa and Prasanth [64]). To further investigate an antibody-borne effect and to verify the spatial proximity of SAF-A with Xist RNA, we transiently transfected C2C12 cells with mouse hrGFP-tagged SAF-A. In line with a previous observation [65], we found SAF-A-hrGFP enriched within the Barr body supporting a possible epitope masking of SAF-A in Barr bodies (Figure 5C). The close spatial proximity between Xist RNA and SAF-A was underpinned by their average minimal distance of approximately 110 to 125 nm in a nearest neighbor analysis (Figure 5D). Of note, control stainings using the same primary antibody simultaneously detected with red and green fluorescent secondary antibodies were measured in a parallel 3D-SIM study and yielded an average minimal distance of approximately 100 nm, which likely represents the collective offset associated with dual-color immunofluorescence detection with 3D-SIM [66] (see Additional file 4).

### Xist RNA shows little spatial proximity with H3K27me3- and macroH2A1-enriched chromatin

Earlier observations described an embedding of Xist RNA in H3K27me3/macroH2A1-enriched chromatin sites along the Xi [67-69] and were recently supported for H3K27me3 by high-resolution molecular analyses [50,68]. However, a direct and stable association of Xist RNA with H3K27me3 or the methylation conferring enzyme complex PRC2 was challenged by other studies (reviewed in Wutz [47], Sengupta *et al*. [59] and Jonkers *et al*. [70]) and further refuted by a recent study from Cerase and co-workers [66]. Here we compared the spatial relationship of Xist RNA with H3K27me3- and macroH2A1-enriched chromatin in the Barr bodies of C2C12 and RPE-1 cells by 3D-SIM after 3D immuno-RNA-FISH. Most Xist RNA foci appeared clearly separated from either H3K27me3- or macroH2A1-labeled chromatin (Additional file 10A,B, left panels) with average minimal distances >150 nm between Xist RNA and H3K27me3 or macroH2A1 signals, respectively (Additional file 10C). Co-immunodetection of macroH2A1- and H3K27me3-labelled chromatin, in contrast, showed a higher level of overlap (Additional file 10, right panels) in line with an average minimal distance of <140 nm between H3K27me3 and macroH2A1 (Additional file 10C; see Additional file 4 for a comparative overview of all assessed minimal distances from this study and Cerase *et al*. [66]). These different spatial proximities were corroborated by respective Manders’ and Pearson’s correlation coefficients (Additional file 11).

### Barr body formation at the onset of XCI in XX ESCs occurs after initial Xist RNA spreading together with RNAP II exclusion

We next studied the localization of Xist RNA at the onset of XCI in early differentiating XX ESCs and followed the process of chromatin compaction towards Barr body formation. Undifferentiated XX ESCs contain two active X chromosomes. Accumulation of Xist RNA at the designated Xi is considered the earliest visible event at the onset of XCI [71,72], reviewed in Heard [1]. A previous study reporting on a gradual exclusion of RNAP II from the ‘Xist RNA domain’ following Xist RNA accumulation found no evidence for chromatin compaction in this domain within an observation time up to day 4 upon differentiation [35].

**Figure 6:**
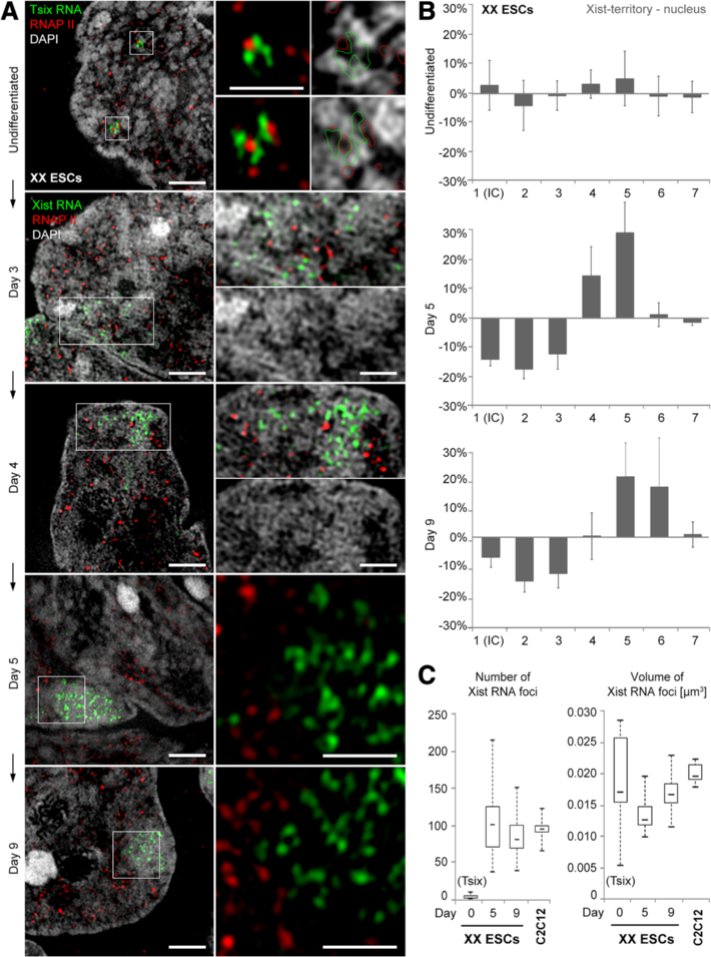
**Barr body formation at onset of XCI in early differentiating female XX ESCs. (A)** Temporal evolvement of the Barr body in relation to Xist RNA spreading and RNAP II exclusion. Undifferentiated: Xist/Tsix RNA transcripts from both X chromosomes before onset of XCI. The fibrillar appearance of RNA signals point to a high local concentration of transcripts around a strong RNAP II signal at each of the two Xist (Tsix) loci. Note that RNAP II and RNA expand into the ANC between the denser packed CDCs. Scale bar: 2 μm, insets 0.5 μm. Day 3: focal spreading of Xist RNA into a confined nuclear region without visible chromatin compaction containing abundant RNAP II signals throughout the loose ‘Xist RNA territory’. Scale bars: 2 μm, insets 1 μm. Day 4: RNAP II signals mostly at the edge of the ‘Xist RNA territory’. Chromatin shows first signs of compaction. Scale bars: 2 μm, insets 1 μm. Day 5 and day 9: Clear outline of a Barr body with distinct chromatin compaction underneath the Xist RNA territory and exclusion of RNAP II. Scale bars: 2 μm, insets 0.5 μm. **(B)** Histograms of DAPI intensity differences (plotted as over/underrepresentation for each class) in the emerging Barr body as compared to the entire nucleus. Mean differences from at least ten nuclei from each state are indicated with standard deviations documenting the shift towards higher intensity classes at day 5 upon differentiation and an additional shift to the right at day 9. **(C)** Boxplots showing the number and volume distributions of discernible Tsix/Xist RNA foci (quantified by Volocity) from single Xi territories in undifferentiated XX ESCs. Day 0, n = 13; day 5, n = 14; and day 9, n = 12. Data for C2C12 (compare Figure 4) are shown for comparison. ANC, active nuclear compartment; CDC, chromatin domain cluster; DAPI, 4',6-diamidino-2-phenylindole; ESC, embryonic stem cell; RNAP II, RNA polymerase II; XCI, X chromosome inactivation; Xi, inactive X chromosome; Xist, X inactive specific transcript.

In the present study we extended the observation period in XX ESCs to identify the time point of Barr body formation during the XCI process. We re-investigated the temporal and spatial correlations between Xist RNA and RNAP II in relation to chromatin compaction in the designated Xi territory by 3D-SIM up to day 9 upon differentiation (Figure 6). In undifferentiated XX ESCs, the Xist probe detected a small RNA cluster on both Xa territories most likely representing Tsix RNA, a non-coding Xist antagonizing RNA, which fully overlaps with the Xist gene and is transcribed in the antisense orientation from both X chromosomes before onset of XCI [73]. These transcripts were found in close association with RNAP II sites, embedded in an overall decondensed chromatin environment and occasionally bridging across ANC channels (Figure 6A). A similar appearance was observed up to day 3 of differentiation. DAPI intensity profiles, recorded from a region with 200 nm radius around Tsix expression sites, were comparable to the profile of entire XX ESC nuclei. Between day 3 and 4 upon differentiation, a fraction of cells had started a pronounced focal spreading of transcripts within an extended though confined nuclear region most likely reflecting Xist RNA expressed from the designated Xi (Figure 6A, day 3). Notably, at this stage RNAP II was found abundant in the emerging ‘Xist RNA territories’ that did not show chromatin compaction. At day 4, when RNAP II sites appeared mostly at the edge of ‘Xist RNA territories’, a consistent chromatin reorganization characteristic for a typical Barr body had not yet occurred (Figure 6A, day 4). At day 5, that is, 1 to 2 days after initial Xist RNA spreading, a typical Barr body, largely depleted from RNAP II sites and clearly demarcated from surrounding less condensed chromatin was observed in the majority of cells. At this stage Xist RNA was distributed throughout, but confined to the newly formed Barr body (Figure 6A,B, day 5). The full level of chromatin compaction in the emerging Barr body, comparable to somatic C2C12 cells, was reached only at day 9 (Figure 6A,B, day 9; compare also Figure 1D). The number and volume measurements of individual Tsix/Xist RNA foci indicated a dynamic behavior during early differentiation (Figure 6C). The high variability between cells in the number of individual Xist RNA foci at day 5 and their consolidation towards smaller numbers and higher volumes at day 9 hint to a multimerization during this early stage of differentiation.

We further observed substantial changes in the H3K27me3 immunostaining pattern during XX ESC differentiation (Additional file 12). In undifferentiated XX ESCs, H3K27me3 was particularly enriched at chromocenters, as previously shown [68]. At an intermediate stage around day 4, the distinct staining of chromocenters became gradually diminished in a large fraction of cells and H3K27me3 signals were distributed over the entire nucleus, slightly enhanced at the nuclear periphery and around nucleoli. Around/after day 5 of differentiation a distinctive marking of the Barr body by a focal H3K27me3 enrichment appeared in a fraction of cells. At this time point these patterns co-existed in parallel within one sample, while the Xi-specific pattern was consistently observed in most cells only after day 7.

### Xist induction in transgenic male ESCs: inconsistent Barr body consolidation and persistent spreading of Xist RNA into decondensed transcriptionally active chromatin

**Figure 7:**
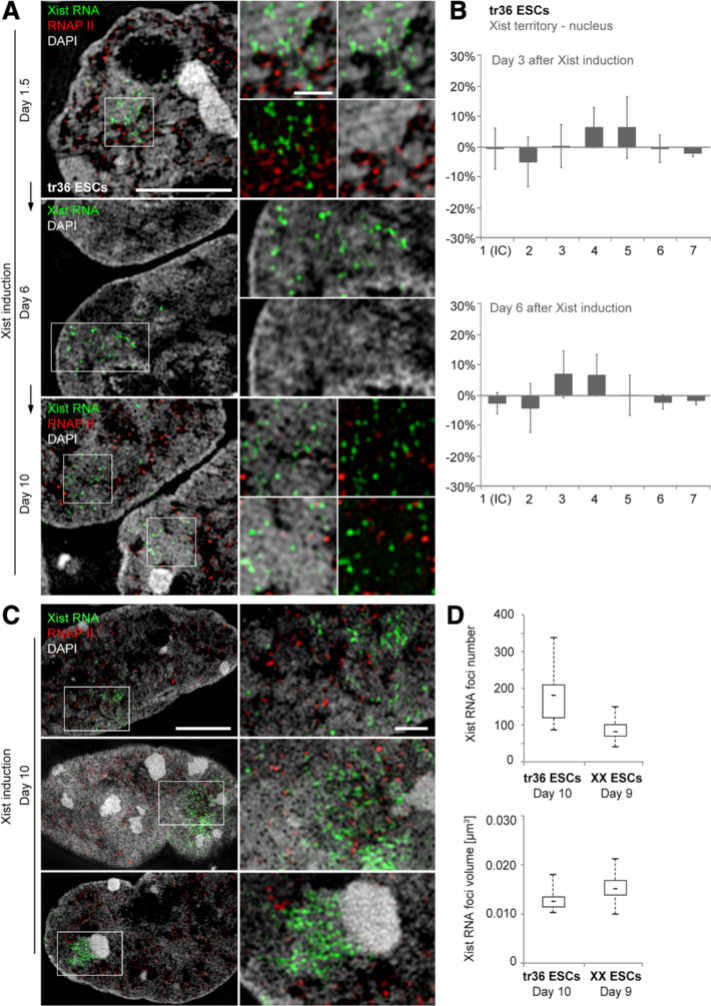
**Failure of ‘autosomal Barr body’ formation consistent with a Xi correlated Barr body after inducing an autosomal Xist transgene. (A)** Immuno-RNA-FISH against Xist RNA (green) and RNAP II (red) at indicated time points after Xist induction. RNAP II is located in close proximity to Xist RNA foci within an extended ‘Xist territory’ at day 1.5. Persistence of RNAP II signals within a widespread ‘Xist territory’ up to day 10 with only slight chromatin compaction visible at days 6 and 10. Scale bars: 5 μm (left column), insets 1 μm (right column). **(B)** Only small changes in DAPI intensity classification of the emerging ‘Xist-territory/autosomal Barr body’ with respect to the entire nucleus 3 and 6 days after Xist induction, plotted as over/underrepresentation. **(C)** Variable manifestations of ‘Xist territories’ 10 days after Xist induction. The top and middle panels exemplify Xist RNA spreading into decondensed chromatin marked by RNAP II, while the bottom panel exemplifies a ‘Xist territory’ with large exclusion of RNAP II. Scale bars: 5 μm, inset 1 μm. **(D)** Number and volume distributions of discernible Xist RNA foci in tr36 ESCs at day 10 of Xist induction (n = 15 nuclei), for comparison shown together with XX ESCs at day 9 of differentiation (see Figure 6). DAPI, 4',6-diamidino-2-phenylindole; ESC, embryonic stem cell; FISH, fluorescence *in situ* hybridization; RNAP II, RNA polymerase II; Xi, inactive X chromosome; Xist, X inactive specific transcript.

We utilized a male mouse ESC line (clone 36, described by Wutz and Jaenisch [49], here termed tr36 ESC) with a doxycycline-inducible Xist transgene stably integrated into chromosome 11 as a model system to study the formation of an ‘autosomal Barr body’. In male ESCs carrying an inducible autosomal Xist transgene, spreading of Xist RNA *in cis* and transcriptional repression of exemplarily examined genes from the respective autosome has been previously demonstrated [49,74,75]. Under our experimental conditions initial spreading of Xist RNA foci in tr36 ESCs occurred approximately 1.5 days after induction within an extended though confined nuclear region, similar to the pattern observed at initial Xist RNA spreading in XX ESCs (Figure 7A). In contrast to XX ESCs, where transformation of the designated Xi into a compacted Barr body, largely congruent with the painted ‘Xist territory’, was accomplished within one or two days after initial Xist spreading, tr36 ESCs failed to form an ‘autosomal Barr body’ fully consistent with an Xi correlated Barr body over an observation period extended up to 10 days. Xist RNA foci in most tr36 ESCs persisted within an expanded nuclear region with diameters up to approximately 4 μm pervading into decondensed chromatin regions. RNAP II signals, still observed within the autosomal ‘Xist territory’ at day 10 after Xist induction indicated the continued potential for transcriptional activity (Figure 7A,B). This phenotype, with variable manifestations of Xist RNA extension into decondensed chromatin marked by RNAP II, was also maintained after differentiation up to an observation period of 10 days (Figure 7C). The amount of discernible Xist RNA foci in tr36 ESCs was over two-fold increased and showed a wider distribution range compared to differentiating XX ESCs (Figure 7D). These features distinguished the ‘autosomal Barr body’ from its X chromosomal counterpart and illustrate its weaker potential for chromatin compaction and transcriptional silencing.

The large spatial expansion of the tr36 ESC ‘Xist territories’ and the high amount of Xist foci made their congruency with a single CT 11 questionable. Xist RNA-FISH combined with painting of CTs 11 showed in fact a wide expansion of Xist RNA beyond painted CTs 11 (Additional file 13A,B). To further clarify this observation we performed a karyotype analysis of tr36 ESCs. Multiplex (M)-FISH revealed a translocation t(11; 11) in 24% and a translocation t(11; autosome) in 5% of analyzed metaphases (Additional file 13C). The increased size of the ‘Xist territory’ and increased number of Xist RNA foci could thus in part be explained by these translocations where Xist RNA would spread onto a larger CT *in cis* represented by the translocation chromosome. The radial spreading of Xist RNA foci several μm beyond painted CTs 11 was, however, unlikely to be explained by the rare observation of a translocation t(11; autosome) and raised the suspicion of Xist RNA diffusing into neighboring CTs in this cell line.

## Discussion

### The Barr body is characterized by a significant collapse of the ANC but maintains principle features of CT architecture

Despite major differences in compaction between transcriptionally competent CTs and the Xi territory, our data imply that both structures share a sponge-like organization, characterized by two spatially contiguous, interacting networks, a higher-order chromatin network built up from compact CDCs and an ANC channel network, which should both be considered as 3D networks with their own dynamic organization. A comparison of H2B-GFP-tagged chromatin in live and fixed cells demonstrated these basic principles of nuclear organization also in nuclei of living cells and ruled out fixation artifacts as a major issue. In addition, DAPI was verified as a suitable marker for global chromatin representation despite its binding preference to AT-rich DNA [52] by a comparison with the DNA sequence-independent dye SYTOX Green.

ANC channels characterized by their reduced DAPI intensities were further substantiated by their connectivity to nuclear pores and by linking topographical DAPI intensity mapping with functionally defined markers. This mapping analysis localized transcription competency markers (H3K4me3, RNAP II) and the nuclear matrix protein SAF-A within or at the boundaries of these channels, while the repressive marker H3K27me3 was strongly overrepresented within higher DAPI intensity classes remote from chromatin boundaries and assigned to interior parts of CDCs.

Our observations support predictions of an extended concept of the chromosome territory-interchromatin compartment (CT-IC) model [17,20-22,29,76]. According to this model, CDCs are composed of approximately 1 Mb CDs carrying transcriptionally silent chromatin localized within the compact core and considered as the inactive nuclear compartment. This compartment is lined by a decondensed periphery of transcriptionally competent chromatin, the PR characterized by small chromatin loops, mostly constrained within a zone of approximately 100 nm, which borders IC channels. These loops are accessible for the assembly of transcription complexes [17,22,26] and represent sites for replication [77], which we show here also for Xi by 3D-SIM (for an experimental proof of principle see Additional file 14). The IC interacts functionally with the PR by providing factors for RNA processing, replication and facilitating nuclear transport. Accordingly, we consider the structurally complex IC/PR as a functionally interacting ANC channel system that can, depending on functional demands, expand or narrow. The figurative term ‘ANC channel’ may be illustrated by comparison with a creek lined by reed beds expanding into the water and not in the sense of a waterway clearly separated from the ‘mainland’ by a concrete embankment.

The concept of CDCs composed of approximately 1 Mb sized chromatin domains was supported by recent population-based Hi-C analyses, a molecular approach to define chromatin proximity patterns at high resolution [18,45,78]. The study of Dixon and co-workers [18] defined this compartmentalized structure as topological domains (TDs) with a median size of several hundred kb, while a small remaining fraction with a size of <50 kb was termed ‘boundary regions’. The similar length scale described for TDs and for microscopically observed 1 Mb chromatin domains suggests that both represent the same structures (reviewed in Gibcus and Dekker [19], Bickmore and van Steensel [79] and Dekker *et al*. [80]). Yet, the extent to which ‘genomic domains’ of a 2D plaid pattern obtained by Hi-C analyses of large cell populations match with the higher-order chromatin landscape perceived for single cells by 3D-SIM cannot be answered conclusively to date. ‘Boundary regions’, particularly enriched in housekeeping genes and active RNAP II [18] might largely represent decondensed chromatin loops in the PR, which may occasionally pervade deeply into the IC.

Our 3D-SIM study suggests that Barr body formation results from a partial collapse of the ANC depending on increased chromatin compaction within the PR and a closer proximity of approximately 1 Mb CDCs/TDs. The clear visualization of low DAPI intensity-defined ANC channels in Xi is compromised due to the small distances between CDCs, which are at or just below the resolution limit of 3D-SIM. Evidence for persisting channels even in the partially collapsed ANC of the Xi is provided by their connectivity to nuclear pores and their opening and expansion under hyperosmolaric conditions. A previous EM study [35] reporting evidence for distinct tunnels that pervade between 200 to 400 nm thick chromatin fibers of the Xi and end at nuclear pores is in line with our observations obtained under hyperosmolaric conditions. However, the width of the collapsed channel system within the Barr body of cells fixed under normotonic conditions seems to be smaller than reported in the EM study.

While the ANC of autosomal CTs is enriched with H3K4me3 and RNAP II, these hallmarks of transcriptionally competent chromatin are scarcely represented in the Barr body. Their occasional occurrence within the Barr body supports recent observations that genes escaping XCI are distributed throughout the Barr body [34,36] and suggests that transcription can also occur within the Barr body. Still, it remains elusive whether the Barr body periphery maintains a more favorable environment for transcription than its interior as previously suggested [32].

### Focal Xist RNA distribution throughout the Barr body at the collapsed ANC channels suggests its dynamic association with silenced genes

Conventional fluorescence microscopy implied the longstanding conception of a cloud of Xist transcripts and a uniform ‘coating’ of the Xi territory [70,81-83]. The organization of Xist RNA as distinct foci distributed throughout the Xi, as revealed by 3D-SIM, may encourage some reconsideration. Notably, as a hypothetical model, a focal organization of Xist was already suggested in 1996 by Clemson and coworkers [84] and further considered in Xist RNA tagging experiments in living ESCs [85]. 3D-SIM also provides an informative basis for the comparative assessment of the number of Xist RNA foci. Our count of less than 100 foci per Barr body both in human and mouse somatic cells was significantly below the estimated 300 to 1,000 copies assessed by qPCR techniques [60,86]. Since a labeled FISH probe bound to one Xist RNA molecule should yield sufficient fluorescence to be detected by 3D-SIM, this discrepancy likely reflects multimerization of Xist RNA molecules [70,87,88] and suggests the aggregation of three to ten Xist RNA transcripts on average for the formation of an individual focus.

Within the 3D chromatin environment of the Barr body, 3D-SIM analyses revealed the preferential localization of Xist RNA foci within and at the boundaries of the collapsed ANC channels. This localization was further elucidated after widening this compartment in the Barr body by induction of HCC. We hypothesize that both the collapsed ANC channels in Xi territories and the open ANC channels in transcriptionally competent CTs are enriched in coding and/or regulatory sequences. In line with this hypothesis RNA foci mark distinct sites of genes or regulatory sequences that become repressed during XCI. The clear spatial separation between most Xist RNA and H3K27me3 signals and a low degree of overlap at the resolution level of 3D-SIM additionally underlines the distinct localization of Xist RNA away from compacted CDCs that are enriched in H3K27me3. Spatial separation of Xist RNA and H3K27me3 is in agreement with our recent 3D-SIM study analyzing Polycomb proteins and Xist RNA [66]. Together, these findings support the recently proposed concept [10], that Xist RNA mediates recruitment of Polycomb proteins via an indirect rather than direct mechanism.

Early studies found Xist RNA enrichment at G-light bands on the Xi in metaphase spreads, suggesting an association of Xist RNA with gene-dense chromatin [89], which was supported by later studies using high-throughput epigenomic mapping [36,50,68,90]. The recent seminal study by Engreitz *et al*. [50] using an RNA antisense purification technology reported on Xist interactions with chromatin independent of sequence specificity. Here, Xist RNA was found to bind broadly across the X chromosome, though enriched at gene-dense sites, in particular at sites of silenced genes. This seeming discrepancy of a distinct focal distribution observed by our 3D-SIM analysis and a rather even Xist RNA distribution found by Engreitz *et al*. may be resolved by considering that the latter approach reflects observations obtained from averaging large cell populations, which may be consistent with a dynamic focal distribution seen at the single-cell level. Taken together this argues for a dynamic association of Xist RNA foci with stochastic binding to a subset of potential chromatin binding sites at a given point in time, rather than to a deterministic stable association at specific sites. These characteristics are evened out in population-based (Hi-C) analyses and our study exemplifies the importance of complementary high-resolution single-cell analyses. Xist RNA might thus contribute to the establishment of a silenced local chromatin environment by inactivating specific regulatory elements suggested by Calabrese *et al*. [36] or by blocking the access for the transcription machinery to the Xi at variable sites.

A structural role of Xist RNA for the maintenance of the specific Xi conformation was recently shown by chromosome conformation capturing and knock-down experiments [15,16], which demonstrated the requirement of Xist RNA for a compacted Xi territory in somatic cells. The longstanding discussion of LINE-1 (L1) repeats as direct anchor points for Xist RNA (for review see Pontier and Gribnau [3]) was contradicted by the recent finding of a negative correlation between Xist RNA and L1 [50]. Yet L1 repeats, overall enriched in the X chromosome and considered to be concentrated in compacted chromatin domains [18,50], may have an indirect impact on Xi compaction. L1 repeats may facilitate the formation of a repressive CC due to their reported tendency of large, repetitive stretches to form stable contacts [78].

### Functional implications of Xist RNA’s spatial proximity to SAF-A

A previous study reported on the requirement of SAF-A for Xist RNA localization to the Barr body [63]. SAF-A has long been known as a nuclear matrix protein with specific DNA binding properties [91] and involvement in transcription, mRNA trafficking and splicing (for review see Han *et al*. [92]). Its potential role in nuclear architecture has remained elusive to date. Our finding of a particulate or fibrous-like SAF-A staining pattern in the ANC of both active CTs and the Barr body supports a function of SAF-A for the structural organization of chromatin within this functional compartment. Evidence for an interaction between Xist RNA and SAF-A was initially based on the dual binding properties of SAF-A to RNA and DNA [93]. RNA immunoprecipitation, enrichment of SAF-A:GFP fusion proteins at the Xi, dissociation of Xist RNA from Xi after SAF-A knock-down and a recently developed *in silico* protein RNA interaction prediction approach made this protein a strong candidate as a Xist RNA interaction factor [63,65]. Yet, a direct spatial interaction between SAF-A and Xist RNA has not been proven so far (reviewed in Nakagawa and Prasanth [64] and Tattermusch and Brockdorff [94]). Our 3D-SIM single-cell analysis revealed a close spatial proximity between Xist RNA and SAF-A. Their functional interaction is further supported by the presumed Xi-specific post-translational modification or conformational switch of SAF-A upon interaction with Xist RNA (reviewed in Nakagawa and Prasanth [64]) resulting in an epitope masking within the Barr body. The resolution limit of 3D-SIM does not allow determination of whether the SAF-A pattern within the collapsed ANC of the Barr body is largely due to tight packing of protein molecules or to a true multimerization. The latter would further argue for a functional role for SAF-A in XCI, since SAF-A multimerization apparently requires interaction with nucleic acids [93]. A binding to Xist RNA could trigger SAF-A multimer formation, creating a scaffold that helps to maintain the integrity of the Barr body structure. Such a function might explain the enrichment of SAF-A within the Barr body.

### Chromatin compaction in the committed Xi of early differentiating XX ESCs is accompanied by RNAP II exclusion at onset of inactivation

Time-resolved 3D-SIM analysis of XCI in XX ESCs revealed initial spreading of Xist RNA foci into a decondensed chromatin environment harboring numerous RNAP II sites around day 3 upon differentiation. Focal Xist RNA spreading was followed by RNAP II exclusion starting the day thereafter, in line with a previous study surveying an observation time up to day 4 [35]. This study showed major exclusion of RNAP II at day 4 but not yet evidence for chromatin compaction. Our extended observation period up to day 10 upon differentiation revealed the distinct global compaction of the Xist RNA-defined Xi territory towards a Barr body typically at day 5. This delay after initial Xist RNA spreading makes it unlikely that Xist RNA acts as an immediate mediator for chromatin compaction of the newly formed Xi. The close temporal occurrence of RNAP II exclusion and Barr body formation suggests a link between Barr body formation and transcriptional repression, but the time-resolution of our differentiation experiments does not allow statements about their temporal order and mutual interdependence, which likely includes other factors as well. It may be speculated that after RNAP II exclusion and/or the removal of H3K4me3 and other active chromatin marks as early events of gene repression during XCI [95], silenced genes in turn quickly undergo chromatin condensation. The higher level of chromatin compaction in the Barr body observed at day 9 may reflect a stable stage of chromatin arrangement in Xi mediated by repressive signatures such as DNA methylation that appear later during XCI in XX ESCs (for reviews see Heard *et al*. [12] and Nora and Heard [96]).

### Structural features of an ‘autosomal Barr body’ differ from its Xi counterpart

Transgenic Xist induction from an autosome in tr36 ESCs failed to consolidate the respective CT into an ‘autosomal Barr body’ with features fully consistent with the Xi-derived Barr body. Spreading of Xist RNA beyond the compacted transgene carrier CT into decondensed, apparently transcriptionally active chromatin was observed up to day 10 upon Xist induction and another 10 days upon differentiation. Thus, this state does not reflect a specific feature of the non-differentiated state. The phenotypic inconsistencies between autosomal and X chromosomal Xist induction add on to the large body of evidence for an impaired silencing efficiency of autosomally transcribed Xist RNA and/or an impaired response of autosomes upon Xist induction, leaving room for different explanations: free Xist RNA may diffuse away from the transgenic autosome, as suggested in Jeon and Lee [83]. Our observation of a large radial expansion of Xist RNA beyond painted CTs 11 may reflect an impaired trapping and transmigration into neighboring CTs. The wide expansion could also reflect spreading *in cis* into abundant, highly extended chromatin loops that were not delineated by chromosome painting. Yet, previous studies found an extensive looping out from the bulk territory restricted to very few regions harboring particular gene-dense and transcriptionally active clusters [20,97-99].

An incomplete Xist RNA-induced gene silencing in the autosomal part of a translocation chromosome t(X; 4) in an XX ESC line was explained by an attenuated spreading of Xist RNA into the autosomal part of the translocation chromosome [100]. The high abundance of Xist RNA foci observed here in tr36 ESCs, however, does not support this assumption. Incomplete inactivation of autosomes has also been considered as a lack of evolutionary adaptation in autosomes, making X chromosomal DNA particularly susceptible for Xist RNA-induced gene silencing possibly by its high enrichment of L1 repeat sequences. Mouse chromosome 11 is an overall L1-poor chromosome, though with a relative enrichment in segments A1 to A5 [53]. One may speculate that compacted chromatin segments largely void of RNAP II seen after transgenic Xist induction in tr36 ESCs might represent chromosome 11 segments enriched in L1 repeats and thus susceptible for gene silencing, while the remaining part of the chromosome may undergo only an incomplete inactivation process.

### Model views of Barr body architecture and general CT architecture shed light on structure-function conundrums of nuclear organization

**Figure 8:**
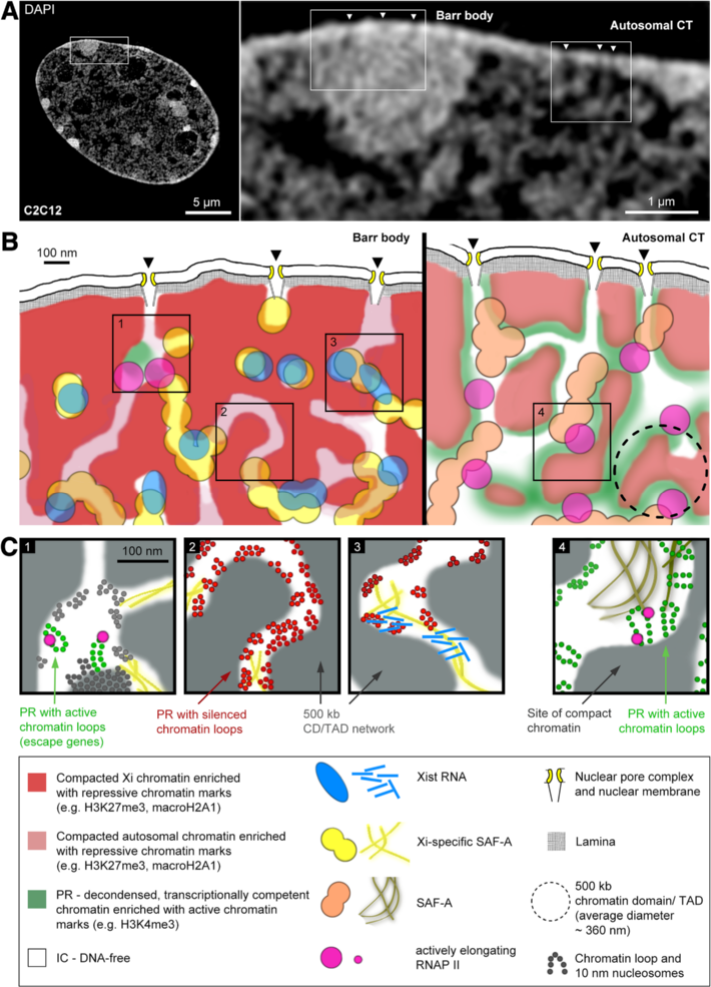
**Model views of Barr body architecture and general CT architecture. (A)** 3D-SIM section through a DAPI-stained C2C12 nucleus with framed areas representing a typical part, both from the Xi and from a neighboring, transcriptionally competent CT. **(B)** To scale scheme of the topographical relationships between chromatin and different targets addressed in this study at 3D-SIM resolution. **(C)** Hypothetical model views of both Xi and autosomal chromatin organization with yet unreached resolution approaching the size of individual nucleosomes. For a detailed description of this figure, refer to the discussion in the main text. 3D-SIM, three-dimensional structured illumination microscopy; CT, chromosome territory; DAPI, 4',6-diamidino-2-phenylindole; Xi, inactive X chromosome.

Model views of the Barr body architecture as the structural hallmark for a transcriptionally repressed CT in comparison to active CTs are presented as virtual 2D sections at different levels of resolution in Figure 8, with the objective to draw them to scale. They integrate our findings on the topographical relationships between chromatin or specific chromatin marks (H3K4me3, H3K27me3) with Xist RNA, SAF-A and RNAP II. The contiguous 3D chromatin network compartment typically consists of CDCs (marked red in Figure 8B), which hamper the identification of individual CDs. Thus, this view argues for a higher-order integration of distinct individual CDs in the order of 500 kb to approximately 1 Mb (encircled in Figure 8B, right) visualized during S-phase as replication foci [101,102] or even smaller subunits reflecting replicons of approximately 150 kb [101]. CD networks also connect neighboring CTs with each other [20,23]. Accordingly, individual CTs are typically not separated by a distinct interchromosome domain as was initially predicted [103]. However, such a margin still holds for nuclei of senescent fibroblasts [104,105] and of bovine embryos [106] emphasizing that there is no one-size-fits-all model of structural interactions or separations of neighboring CTs.

In autosomal CTs (Figure 8B, right), the transcriptionally competent periphery of CDCs, that is the PR (colored in green), signifies its enrichment in active histone marks confined within a zone of approximately 100 nm around segmented chromatin borders. Chromatin domains may co-exist, which either represent transcriptionally competent or silent chromatin [18,45]. The local shape and width of both CDCs and the IC are highly variable, with larger IC lacunae harboring nuclear bodies [29] (not shown here). The IC channel system carries SAF-A, indicating a nuclear matrix expanding within the IC. Whether the interior of the IC is actually free of chromatin cannot conclusively be assessed on DAPI-stained 3D-SIM images, since they do not provide the resolution to detect individual DNA molecules within the context of a bulk DNA/chromatin staining. EM evidence based on the specific display of DNA, however, provide strong substantiation for a general absence of many chromatin loops expanding into the interior of wider IC channels and lacunae [23].

Compared to autosomal CTs, H3K27me3-enriched CDCs within the Barr body are moved closer together. A higher compaction of individual CDs can be assumed but is, again, beyond the resolution limit of 3D-SIM. ANC channels appear significantly collapsed to the extent that only a few non-collapsed sites are detectable that may harbor active chromatin marks (Figure 8B, left). Xist RNA foci are found along the collapsed ANC.

Figure 8C provides speculative model views at nucleosomal resolution, co-existing within a Barr body (boxes 1, 2 and 3) and within a transcriptionally active region typical for an autosomal CT (box 4). They are drawn as experimentally testable predictions for future studies considering the potential 3D organization of chromatin at the resolution level of the approximate 10 nm nucleosome fiber. Box 1 illustrates transcriptional activity of escaped genes in the Barr body interior, supported by the finding of escaped genes throughout the Barr body [34]. In line with the structural organization of transcriptionally competent sites in autosomal CTs (box 4), we propose that transcriptional activity requires small extended 10 nm thick chromatin loops. These loops are enriched in active histone marks and expand from the compacted core chromatin into the ANC facilitating biochemical interactions with nuclear bodies, recruitment of factors required for transcription and for co-transcriptional splicing. Of note, extended chromatin loops comprise only a few kb, much below the size of many genes and thus require a highly dynamic organization of chromatin domains for the transcription of an entire gene since only a small part of it is actually presented as an expanded loop where transcription initiation complexes and chromatin remodeling factors can bind. A transcribed part of a gene is rapidly re-compacted. Similar scenarios can be envisaged for chromatin replication and DNA repair implicating the requirement for a dynamic organization also at transcriptionally silent locations. Pulse DNA replication labeling experiments with varying chase times after incorporation of labeled nucleotides demonstrate the location of nascent DNA at boundaries of CDCs, whereas with increasing chase time post-replicative DNA is progressively shifted into the cluster interior (see Additional file 14 and Fakan and van Driel [26] for previous TEM data). Boxes 2 and 3 exemplify hypothetical scenarios for transcriptionally silent regions within the Barr body. Less condensed chromatin is present in the collapsed ANC in the form of ‘closed’ configurations of 10 nm thick chromatin fibers, possibly enriched with repressive histone marks. At some sites a narrow, chromatin-free IC channel may exist, while other sites are filled completely with ‘closed’ or ‘open’ chromatin loops needed for replication or repair tasks. Box 3 illustrates the possibility of fully compacted neighboring CDCs without any chromatin looping into the narrow IC channel. Xist RNAs accumulate at specific yet DNA sequence-independent sites, where they may dynamically interact with XCI-susceptible genes/regulatory sequences, clogging the IC channel at this site and using Xi-specific SAF-A as an anchor which may also be essential for a structural separation of CDCs.

Even in a transcriptionally fully silent part of a CT, it is essential that the space-time organization of CDs/TDs is compatible with their ‘opening’ and ‘closure’ to fulfill the demands of chromatin replication and DNA repair. Maintenance of a compartmentalization in the Barr body may be important to avoid chromatin entanglement, which could interfere with necessary chromatin movements during chromatin replication and the structural transformation into a mitotic chromosome [107].

## Conclusions

Barr body formation of the committed Xi at onset of inactivation occurs with a >1 day delay after initial Xist RNA spreading in close temporal connection with subsequent RNAP II exclusion suggesting an interdependence between transcriptional repression and chromatin compaction.

3D SIM shows that the Barr body maintains principle ultrastructural features of a general CT architecture, which consists of a network of CDCs with a compact chromatin core lined by a zone of decondensed transcriptionally permissive chromatin, the PR. CDCs are pervaded by an IC channel system, which is connected to nuclear pores and forms together with the PR the ANC, where transcription and chromatin replication occurs. In the Barr body the ANC appears partially collapsed. The observation of Xist RNA foci within the partially collapsed ANC supports the conception of Xist RNA interacting with chromatin representing genes or regulatory elements. The appearance of approximately 60 (RPE-1) and 100 (C2C12) distinct Xist RNA foci seen within an individual Barr body may represent a snap-shot of a dynamic interaction of these foci with silenced genes located within the collapsed ANC. Enrichment of the nuclear matrix protein SAF-A within Xi and its close spatial association with Xist RNA foci suggests a function of this ‘complex’ for structural organization of Xi. The structural maintenance of CDCs and an albeit rudimentary ANC channel system with connections to nuclear pores in the Barr body may be a requirement for sparse transcription and proper replication of the Barr body.

## Methods

### Cells and culture conditions

Human retina epithelial-derived, hTERT-RPE-1 cells were grown in DMEM/HF-12 (1:1) supplemented with 10% FCS. They were characterized as tetraploid with few chromosomal rearrangements (M-FISH data upon request). Female mouse C2C12 myoblast cells, cultured in DMEM/20% FCS, are near tetraploid, including four copies of the X chromosome [108]. Both cell lines contain two inactive X chromosomes. HeLa cells stably transfected with histone H2B-GFP [109] were grown in RPMI 1640/10% FCS.

For transient transfection, RPE-1 and C2C12 cells were grown in six-well dishes up to 70% confluency and transfected with 1 μg of pBOS_H2B-GFP, pCAGGS_SAF-Ah:hrGFP or pCAGGS_SAF-Am:hrGFP (human and mouse SAF-A, respectively). Transfections were done with Lipofectamine 2000 (Invitrogen, Carlsbad, CA, USA) following the supplier’s instructions. Cells were fixed 24 h post-transfection. For induction of HCC, cells were incubated for 5 min in hyperosmolaric (approximately 750 mOsm) medium before fixation as previously described [20,62]. For pulse replication labeling by incorporation of the thymidine analogue 5-ethynyl-2-deoxyuridine (EdU), EdU was added at a final concentration of 10 μM to the medium for 5 min.

Undifferentiated mouse ESC lines, the female wild type ESC (clone E16.7, XX ESCs) [110] and the male Xist-transgenic ESC (J1 ESC, clone 36, tr36 ESCs) [49] containing one copy of full-length mouse Xist cDNA under the control of a doxycycline inducible promoter on chromosome 11 were cultivated under feeder-free conditions on gelatinized cover slips in KO-DMEM (Invitrogen) supplemented with 16% FBS (stem cell tested; GE-Healthcare, Little Chalfont, UK), 1% non-essential amino acids (100 × stock; PAA), 2 mM GlutaMAX (Invitrogen) and 0.1 mM β-mercaptoethanol (Roth, Karlsruhe; Germany). To maintain the undifferentiated state, culture media were supplemented with 1,000 U/ml LIF (PAA), 1 μM PD 0325901 (MEK inhibitor; Axon Medchem, Groningen, The Netherlands) and 3 μM CHIR 99021 (CSK3 inhibitor; Axon Medchem). Medium was changed daily and cells were split every second day. The undifferentiated state was monitored by the typical morphology of ESC colonies as well as by immunodetection of SSEA-1 (Millipore, Billerica, MA, USA). After differentiation, induced by withdrawal of LIF, PD 0325901 and CHIR 99021, cells were cultivated for up to 10 days.

Xist transcription in tr36 ESCs was induced by adding 1 μg/ml doxycycline to the medium. XX ESCs have a diploid karyotype 42,XX,+6,+8 (M-FISH data on request).

### 3D DNA/RNA-FISH, immunodetection and immuno-FISH

Chromosome painting probes delineating human chromosome X or mouse chromosomes 11 and X, generated from flow sorted chromosomes (gift of M Ferguson-Smith, University of Cambridge, Cambridge, UK) were amplified and hapten-labeled by degenerate oligonucleotide-primed (DOP)-PCR using the 6 MW primer as previously described in detail [111]. Next, 40 ng of labeled probe was used per μl of hybridization mix. Fixation and pretreatment steps of cells for DNA-FISH were performed as previously described [48]. For delineation of human Xist transcripts, a full-length cDNA (OriGene, Rockville, MD, USA) was used and amplified by a whole genome amplification kit (Genomi-Phi; GE Healthcare, Fairfield, CT, USA). Mouse Xist RNA probes were generated by specific amplification of exons 1a and 6. Xist RNA probes were labeled with biotin by nick translation. Then, 20 ng of labeled Xist RNA probe and 100 ng salmon sperm DNA were dissolved per μl of hybridization solution (50% formamide/2 × SSC/10% dextran sulfate). Ribonucleoside vanadyl complex (New England Biolabs, Ipswich, MA, USA) was added to the permeabilization buffer and to the probe at 2 mM final concentration to prevent RNase activity.

The following antibodies were used for immunodetection: antibodies against RNAP II Ser2P, detecting the actively elongating form of RNAP II [112], (rat monoclonal; kindly provided by D Eick, Ludwig Maximilians University (LMU) Munich, Munich, Germany), H3K27me3 (mouse monoclonal; Active Motif, Carlsbad, CA, USA), macroH2A1 (rabbit polyclonal; Active Motif), PCNA (rat monoclonal; Heinrich Leonhardt Lab, Martinsried, Germany), murine SAF-A (rabbit polyclonal; Brockdorff Lab, Oxford, UK) and hrGFP (polyclonal; Agilent Technologies, Santa Clara, CA, USA). Prior to using the SAF-A antibodies in the experiments described here they were tested by immunofluorescence on a variety of cell lines, antibody-antigen competition assays and immunoprecipitation followed by either Western blotting or mass spectrometry.

For immunofluorescence (IF) detection, cells were seeded on 18 × 18 mm borosilicate glass coverslips, number 1.5H (170 μm ± 5 μm thickness; Marienfeld Superior, Lauda-Königshofen, Germany). Cells were washed two times with PBS and fixed with 2% formaldehyde/PBS for 10 min following a stepwise replacement with 0.05% PBS/Tween (PBST). For permeabilization, cells were incubated in 0.5% Triton X-100/PBS for 10 min and subsequently washed twice in PBST.

For combined immuno-RNA-FISH, cells were equilibrated in 2 × SSC and incubated in 50% formamide/2 × SSC at 4°C for 2 to 4 h. Labeled and denatured RNA-FISH probe was added, cells were mounted on slides, sealed with removable rubber cement and samples were allowed to hybridize at 37°C overnight. Unbound probes were removed with 3 × washing in 2 × SCC and 3 × washing with 4 × SSCT at 37°C and probe detection was carried out in 2% BSA/0.5% FSG/4 × SSCT for 1 h at room temperature.

For subsequent IF, cells were equilibrated in 1 × PBST and blocked with 2% BSA/0.5% FSG/PBST for 1 h. Antibodies were diluted in blocking solution and incubated for 1 h followed by thorough washing with PBST. For detailed description of the immuno-RNA-FISH procedure for super-resolution microscopy see Markaki *et al*. [48]. After the IF procedure, cells were post-fixed for 10 min in 4% formaldehyde/PBS. DNA was counterstained with 1 μg/ml DAPI or 0.25 μM SYTOX Green (Molecular Probes, Eugene, OR, USA) in PBS for 10 min. Samples were mounted in Vectashield antifade mounting medium (Vector Laboratories, Burlingame, CA, USA) and sealed with nail varnish. In case of a combined RNA-/DNA-FISH approach, positions of individual cells were stored and imaged subsequently as described in detail by Markaki *et al*. [48].

### 3D-SIM

Super-resolution imaging on fixed samples was performed on a DeltaVision OMX V3 system (Applied Precision, GE Healthcare) equipped with a 100 ×/1.40 NA Plan Apo oil immersion objective (Olympus, Tokyo, Japan), Cascade II:512 EMCCD cameras (Photometrics, Tucson, AZ, USA) and 405, 488 and 593 nm diode lasers [113]. Live-cell super-resolution imaging was performed with a DeltaVision OMX V3 Blaze system (Applied Precision), equipped with a 60 ×/1.42 NA Plan Apo oil objective and Olympus and sCMOS cameras (PCO, Kelheim, Germany) for high-speed stack acquisition. In both cases, 3D-SIM image stacks were acquired with a z-distance of 125 nm and with 15 raw images per plane (five phases, three angles). The raw data was then computationally reconstructed using Wiener filter settings 0.002 and channel-specifically measured optical transfer functions (OTFs) using the softWoRx 6.0 software package (Applied Precision) to obtain a super-resolution 3D image stack with a lateral (x-y) resolution of approximately 120 nm and an axial (z) resolution of approximately 300 nm [31,43]. The level of spherical aberration was minimized and matched to the respective OTFs using immersion oil different refractive indices (RIs). Best results were typically obtained with OTFs measured on 110 nm diameter red and green fluorescent FluoSpheres (Invitrogen) and 170 nm diameter blue fluorescent FluoSpheres (Invitrogen), respectively, using RI 1.512, and sample acquisition with RI 1.514 for depth adjustment in the region of optimal reconstruction a few μm into the sample. Images from the different color channels were registered with alignment parameters obtained from calibration measurements with 0.2 μm diameter TetraSpeck beads (Invitrogen). The reconstruction process generates 32-bit data sets with the pixel number doubled in the lateral axes, and the lateral pixel size halved from 80 nm to 40 nm in order to meet the Nyquist sampling criterion.

To normalize all image stacks for subsequent image processing and data analysis, the stack-specific mode grey value (representing the peak of the background noise) was subtracted, negative values discarded and finally the format converted to 16-bit composite TIFF stacks using an in-house script in ImageJ (http://rsbweb.nih.gov/ij). In some cases 32-bit images were first shifted to positive values, prior to 16-bit transformation and subsequent mode subtraction, leading to identical results.

Conventional wide-field (deconvolution) image stacks were generated from 3D-SIM raw data by average projection of five consecutive phase-shifted images from each plane (only of the first angle) and, in case, subjected it to an iterative 3D deconvolution using softWoRx 6.0. For direct comparison with 3D-SIM images, the pixel numbers were doubled in x and y using a bicubic interpolation in ImageJ to unify voxel sizes in all cases to 40 × 40 × 125 nm.

### Chromatin density classification by 3D assessment of DAPI/SYTOX Green intensity classes

For chromatin density quantification, a hidden Markov random field model classification, combining a finite Gaussian mixture model with a spatial model (Potts model) was used, implemented in the open-source statistics software R [114]. Fluorescently stained DNA was segmented into seven classes with equal intensity variance. This approach allows threshold-independent signal intensity classification at the voxel level, not only based on the intensity of an individual voxel but also considering the classification of surrounding voxels (for a detailed description see Zhang *et al*. [115]). Class 1 represents voxels with intensities close to background level, while class 7 assigns highest chromatin ‘density’. This approach compensates for DNA/chromatin staining intensity variations between individual cells, cell types and experiments. Prior to segmentation, a 3D mask was generated in ImageJ to define the nuclear space according to the DAPI signal. Nucleoli contributed with <5% to the total nuclear volume in both C2C12 and RPE-1 cells (data not shown) and were included in DAPI intensity classifications. 3D masks for Barr bodies, defined as volumes with an enrichment of Xist RNA, were generated by Otsu thresholding of the Xist RNA signals followed by transformation into a binary mask file and dilation by several pixels.

### Quantification of 3D-SIM discernible segmented Xist RNA objects and colocalization analyses

Number and volumes of Xist RNA foci were quantified using Volocity (Perkin Elmer, Waltham, MA, USA). Objects were defined by the ‘separate touching objects’ function after setting an intensity threshold monitored by visual inspection of the signals’ intensity histogram. 3D renderings were performed with Amira 5.2.2 (Visualization Sciences Group, Burlington, MA, USA). In order to make DAPI signals comparable between different nuclei, the DAPI intensities were leveled to the same mean value.

Colocalization analyses were based on Manders’ coefficients M1 and M2, quantifying the amount of overlapping pixels, and Pearson’s correlation (PC) coefficient, assessing the correlation of data sets in a voxel-by-voxel intensity-based analysis. Pearson’s coefficients were calculated on non-thresholded images from the fraction of the stack containing the Barr body volume (approximately 15 z stacks) and its surroundings, or similar sized volumes of the controls, respectively. Barr bodies were defined as volumes with an enrichment of Xist RNA including their close surroundings. The ‘Barr body’ 3D mask was obtained by applying a Gaussian filter, thresholding to remove low intensity signals and converting the obtained stack into a binary file which mapped all voxels of interest for coefficient calculation. Manders’ coefficients were calculated for the signal intensities of voxels in a similar way. To estimate the threshold, for every image stack for both channels a separate small 3D volume from the area outside the cell or nucleus was selected. Average intensity of this ‘background sub-stack’ was calculated and served as a base to calculate the threshold for Manders’ coefficient calculation.

### Nearest neighbor analysis and quantitative localization of specific nuclear targets correlated to DAPI intensity classes

Nearest neighbor/minimal distance measurements were performed using the TANGO Plugin for ImageJ/Fiji [116]. Mode subtracted, 16-bit transformed 3D-SIM image stacks were imported into TANGO. Nuclear masks were generated from the DAPI channel using a watershed algorithm to segment the signals from background and morphological filters to transform the signals to coherent binary mask, covering the entire nuclear space. Barr body masks were generated as described above by dilating the Xist signals covering the Barr body region. For spot centroid determination, the signals in the red and green channels were pre-filtered and segmented as follows: 1) top hat filter with one pixel radius in x-y and z; 2) Laplacian of Gauss filter with one pixel radius in x-y and z; and 3) spot detector 3D with Otsu auto-thresholding. The segmented objects were discarded if their signal intensity was less than twice the mean intensity of the image and if their volume was smaller than two voxels. Finally the minimal distances of intensity weighted centroid xyz-positions of all segmented green and red signals/spots within the masked nuclear or Barr body region were determined. Statistical differences in colocalization coefficients as well as nearest neighbor distances of different experiments were analyzed by pairwise *t*-test comparison with Bonferroni correction of level of significance. To determine the distribution of defined nuclear targets with regard to chromatin intensity classes the centroid xyz-coordinates of TANGO-segmented objects were mapped on the segmented chromatin classes obtained as described above. For calculating the over/underrepresentation of target signals in each chromatin class, the respective fraction sizes were subtracted for each nucleus/Barr body, and the mean values and standard deviations determined. Alternatively, over/underrepresentation was normalized for the chromatin class size by calculating the ratio between target signal fraction and chromatin class fraction and subtracting the value 1 (for a workflow see Additional file 15).

## Abbreviations

1.5: IQR 1.5 × Interquartile range; 3D: Three-dimensional; 3D-SIM: Three-dimensional structured illumination microscopy; ANC: Active nuclear compartment; BSA: Bovine serum albumin; CC: Chromatin compartment; CD: Chromatin domain; CDC: Chromatin domain cluster; CT: Chromosome territory; DAPI: 4',6-Diamidino-2-phenylindole; DMEM: Dulbecco’s modified Eagle’s medium; DOP: Degenerate oligonucleotide-primed; EdU: 5-Ethynyl-2-deoxyuridine; EM: Electron microscopy; ESC: Embryonic stem cell; FCS: Fetal calf serum; FISH: fluorescence *in situ* hybridization; GFP: Green fluorescent protein; H3K27me3: Trimethylated histone H3 lysine 27; H3K4me3: Trimethylated histone H3 lysine 4; HCC: Hypercondensed chromatin; IC: Interchromatin compartment; IF: Immunofluorescence; L1: LINE-1; LMU: Ludwig Maximilians University; Mb: Megabase; OTF: Optical transfer function; PCR: Polymerase chain reaction; PR: Perichromatin region; PRC2: Polycomb recruitment complex 2; qPCR: Quantitative polymerase chain reaction; RI: Refractive index; RNAP: II RNA polymerase II; SAF-A: Scaffold attachment factor-A; TD: Topological domain; TEM: Transmission electron microscopic; tr36: ESC Male embryonic stem cell with Xist transgene in chromosome 11; Xa: Active X chromosome; XCI: X chromosome inactivation; Xi: Inactive X chromosome; Xist: X inactive specific transcript; XX: ESC Female embryonic stem cell.

## Competing interests

The authors declare that they have no competing interests.

## Authors’ contributions

DS performed 3D immuno-FISH and live-cell experiments, image recording and quantitative data analysis, participated in the design of the study, and helped to draft the manuscript. YM performed 3D immuno-FISH experiments, image recording and image analysis, and participated in the design of the study and critical revision of the manuscript. VJS developed and provided analysis tools for quantitative image analysis and performed quantitative data analysis. FK performed quantitative data analysis and statistical analysis. AT generated and characterized SAF-A antibodies and cloned SAF-A constructs. AC contributed to immuno-FISH experiments. MS performed immunodetection experiments and image recording on ES cells. SF performed replication pulse labeling experiments. JD performed live-cell/SYTOX 3D-SIM experiments. JP contributed with data evaluation and drafting the model figure. HL discussed experimental strategy and data interpretation. NL participated in the design of the study and in critical revision of the manuscript draft. TC designed the study, made substantial contributions to conception and participated in writing the manuscript. LS contributed to design of the study, conceived data recording and analysis, and contributed to writing the manuscript. MC conceived the study, performed experiments and essentially wrote the manuscript. All authors provided comments to the draft manuscript, and read and approved the final manuscript.

## Supplementary Material

Additional file 1 **3D-SIM-based DAPI intensity classification in the Barr body versus the entire nucleus of RPE-1 cells. (A)** Mid z-section of a DAPI-stained nucleus. The area below the dashed line illustrates the resolution level obtained by wide-field deconvolution microscopy, for comparison*.* Inset magnifications of the framed areas show the non-uniformly compacted structure of the Barr body resolvable with 3D-SIM (1) and an arbitrary autosomal region with CDCs (2). Scale bars: 5 μm, insets 1 μm. **(B)** X chromosome-specific painting (green) of Xi (left) and Xa territories (right) of the same nucleus in different z-sections. .Scale bars: 2 μm, insets 1 μm. **(C)** 3D DAPI intensity classification exemplified for the nucleus shown in (A). Seven DAPI intensity classes are displayed in false-color code ranging from class 1 (blue) representing pixels close to background intensity, largely representing the IC, up to class 7 (white) representing pixels with the highest density. Framed areas of the Barr body region (inset 1) and a representative autosomal region (inset 2) are shown as magnified on the right at the resolution levels of 3D-SIM, deconvolution and conventional wide-field microscopy. The non-uniformity of the Xi territory pervaded by areas of lower DAPI intensity classes becomes evident at 3D-SIM resolution, whereas both wide-field and deconvolution microscopy imply a concentric increase of density. In the representative autosomal region chromatin assigned to classes 2 to 3 lines compacted CDCs, represented by classes 4 to 6. **(D)** Left panel: average DAPI intensity classification profiles and respective standard deviations obtained from evaluation of entire nuclear volumes (n = 30 nuclei) or the Barr body region only (dark grey bars, n = 26 Barr bodies). Right panel: over/underrepresentation of the average DAPI intensity class fraction sizes in the Barr body versus entire nuclear volumes. 3D-SIM, three-dimensional structured illumination microscopy; CDC, chromatin domain cluster; DAPI, 4',6-diamidino-2-phenylindole; FISH, fluorescence *in situ* hybridization; IC, interchromatin compartment; Xa, active X chromosome; Xi, inactive X chromosome. Click here for file

Additional file 2 **Comparison of SYTOX, H2B-GFP and DAPI staining pattern by 3D-SIM. (A)** Optical section of a DAPI- and SYTOX-stained C2C12 nucleus confirms the similar organization of compact CDCs, a high congruency of domain surfaces even at sites of decondensed chromatin and an IC channel system with a significant narrowing of the channels in the Barr body both after SYTOX and DAPI staining. The distinctly higher intensity of chromocenters in DAPI stains reflects their particularly high AT content. Inset magnifications show arbitrary autosomal regions (1) and the Barr body region (2). Scale bars: 5 μm, insets 1 μm. **(B)** Comparing histograms of DAPI and SYTOX intensity classification profiles for C2C12 and RPE-1 whole nuclei demonstrate similar intensity class distributions. **(C)** Optical section of a histone H2B-GFP expressed in a C2C12 cell nucleus shows, similar to DAPI, an organization of compact CDCs and an IC channel compartment that is narrowed in the Xi territory. Inset magnifications show an arbitrary autosomal region (inset 1) and the Xi territory (inset 2) as identified by H3K27me3 immunolabeling (not shown). Differences in the conformity of domain surfaces may in part be due to (cell cycle-dependent) uptake kinetics of transient H2B-GFP transfection. **(D)** Comparison of H2B-GFP stably expressed in an ESC cell line (green) and DAPI (magenta) staining together with RNAP II (grey) demonstrate a high conformity both at sites of decondensed and condensed chromatin. Scale bar: 1 μm. 3D-SIM, three-dimensional structured illumination microscopy; CDC, chromatin domain cluster; DAPI, 4',6-diamidino-2-phenylindole; ESC, embryonic stem cell; GFP, green fluorescent protein; H3K27me3, trimethylated histone H3 lysine 27; IC, interchromatin compartment; RNAP II, RNA polymerase II [52,117-120]. Click here for file

Additional file 3 **DAPI intensity classification of painted Xa and Xi territories after 3D-FISH.** The average AT content is approximately 59% in the human and 58% in the mouse genome with individual chromosomes ranging from approximately 52 to 63% in humans and 56 to 61% in mouse. With an AT content of approximately 61% in both human and mouse the X chromosome represents (the most) AT-rich chromosome [53]. It might thus be expected that X territories bind DAPI above average and therefore appear to have an increased compaction also on Xa. We therefore painted X territories in C2C12 cells by 3D-FISH and assessed DAPI intensity classification underneath the painted territories (as shown in Figure 1B). Density distributions within Xa territories were in fact intermediate between the entire nucleus and the Xi in line with the relatively high AT content of the X chromosome and possibly also with its overall low gene density and resulting low transcriptional activity (http://www.ncbi.nlm.nih.gov/mapview). The hybridized probe likely contributes in addition towards higher DAPI intensities underneath painted territories since DAPI fluorescence increases significantly when it is bound to double-stranded DNA [52], while the remaining nucleus may still harbor a significant fraction of single stranded DNA after denaturation. The histogram displays the under/overrepresentation of DAPI intensity classes relative to the entire nuclear chromatin region in painted Xa (light grey) and Xi territories (dark grey). Error bars mark the standard deviations. *P* <0.001 (n = 5 cells). DAPI, 4',6-diamidino-2-phenylindole; FISH, fluorescence *in situ* hybridization; Xa, active X chromosome; Xi, inactive X chromosome. Click here for file

Additional file 4 **Overview of nearest neighbor measurements.** Distance measurements between histone markers (indicated by blue bars), between Xist RNA and histone markers (yellow bars), Xist RNA and SAF-A (red bars) were performed in this study. Control measurements (grey bars) were taken from a parallel study [66]. **(A)** Minimal distance distributions combined from all cells with their medians displayed as box plots (median, Q1, Q3) with whiskers indicating the 1.5 IQR. **(B)** Mean of the mean values obtained for individual cells with their respective error (SEM) are displayed demonstrating a very small variation between individual cell measurements. **(C)** Fraction of nearest neighbor distances below 100 nm. **(D)** List of all distance measurements performed in this study and in a parallel study [66]. As ‘colocalization’ control mouse fibroblasts were labeled with primary antibodies against H3K27me3, which were simultaneously detected with two secondary antibody species conjugated to different dyes. Their average minimal distance was approximately 100 nm with 60% of minimal distances below 100 nm, which reflects the collective ‘error’ of the applied method due to detection/labeling specificity, optical mismatch and evaluation inaccuracies (discussed in detail in Cerase *et al*. [66]). 1.5 IQR, 1.5 × interquartile range; H3K27me3, trimethylated histone H3 lysine 27; SAF-A, scaffold attachment factor-A; SEM, standard error of the mean; Xist, X inactive specific transcript. Click here for file

Additional file 5 **Movie 1.** Serial orthogonal sections from the 3D reconstructions in Figure 2. Boxes indicate volume containing the Barr body (right) and neighboring autosomal chromatin (left) with DAPI (red) and inverted DAPI signal (ANC; green) together with an IF staining against Nup153 (blue). ANC, active nuclear compartment; DAPI, 4',6-diamidino-2-phenylindole; IF, immunofluorescence. Click here for file

Additional file 6 **Movie 2.** Same volume from the same nucleus as Movie 1 showing only the ANC (green). ANC, active nuclear compartment. Click here for file

Additional file 7 **Movie 3.** Same volume from the same nucleus as Movie 1 displayed together with nuclear pores (blue). Click here for file

Additional file 8 **Super-resolution imaging of histone H2B-GFP in living and fixed cells. (A)** HeLa cells stably expressing histone H2B-GFP imaged in the living state (upper panel) and after formaldehyde fixation (lower panel). Basic features of chromatin organization, for example compact CDCs, sites of decondensed chromatin, IC (lacunae) leading to nuclear pores, are similar between living and fixed cells. Scale bar: 5 μm, insets 1 μm. **(B)** Comparison of H2B-GFP (green) and DAPI (magenta) staining to evaluate DAPI’s overall chromatin coverage capacity and its general suitability as a marker of chromatin after fixation in formaldehyde, permeabilization and mounting in Vectashield. DAPI and H2B-GFP show a high conformity even at sites of decondensed chromatin or at chromatin voids at nuclear pores. Scale bar: 5 μm, insets 1 μm. CDC, chromatin domain cluster; DAPI, 4',6-diamidino-2-phenylindole; GFP, green fluorescent protein; IC, interchromatin compartment. Click here for file

Additional file 9 **Detailed illustration of Xist RNA localization within low DAPI intensity sites. (A)** DAPI intensity profile in a mid z-section through the Barr body of a DAPI-stained C2C12 nucleus after classification of the entire nucleus. Right panel shows Xist RNA foci (green) in the respective section after conversion of classified DAPI intensities into greyscale. **(B)** Same nucleus after classification only of the Barr body unravels a broader stretching and more detailed chromatin classification. Overlay with the Xist RNA signal depicts the preferential localization of Xist RNA within low chromatin density classes (right). DAPI, 4',6-diamidino-2-phenylindole; Xist, X inactive specific transcript. Click here for file

Additional file 10 **Spatial association of Xist RNA with H3K27me3- and macroH2A1-marked chromatin in RPE-1 and C2C12 nuclei.** Mid z-sections of DAPI-stained **(A)** RPE-1 and **(B)** C2C12 nuclei after immuno-RNA-FISH against Xist RNA (green) and H3K27me3 (red), Xist RNA and macroH2A1 (red), or immunodetection of H3K27me3 (green) and macroH2A1 (red). Inset magnifications of depicted areas demonstrate the distinct localization of Xist RNA from both H3K27me3 and macroH2A1 and a partial overlap between H3K27me3 and macroH2A1. Scale bar: 5 μm, insets 1 μm. **(C)** Boxplots of minimal distance distributions between Xist RNA signals and H3K27me3 or mH2A1 as well as between H3K27me3 or mH2A1. DAPI, 4',6-diamidino-2-phenylindole; FISH, fluorescence *in situ* hybridization; H3K27me3, trimethylated histone H3 lysine 27; Xist, X inactive specific transcript. Click here for file

Additional file 11 **Colocalization analysis using Pearson’s and Manders’ correlation coefficients. (A)** Quantification of colocalization between H3K27me3 and macroH2A1 with Xist RNA in C2C12 (left column) and RPE-1 cells (right column) using Manders’ (M1 and M2) and Pearson’s correlation (PC) coefficients (n = 10 nuclei per evaluation). **(B)** Colocalization coefficients become more stringent with increasing optical resolution. To link visual impression, numerical colocalization values and absolute distances obtained by 3D-SIM, different degrees of (partial) colocalization were simulated in an idealized example. One single greyscale image showing objects in the size range of approximately 100 to 200 nm was copied into two color channels (green and red, distance = 0 nm; complete overlap = yellow; Pearson’s coefficient (PC) = 1.0; Manders’ coefficient (M1) = 1.0). Subsequent shifts of the red against the green channel at indicated lengths in x-direction reveal visual separation of the differently colored objects at distances of >40 nm. A first clear separation of the two channels is seen at distances of approximately 80 nm, while shifting of 160 nm shows a clear gap between the signals of the two channels. PC as well as M1 coefficients are indicated at each step. Scale bar: 0.5 μm. 3D-SIM, three-dimensional structured illumination microscopy; H3K27me3, trimethylated histone H3 lysine 27; PC, Pearson’s correlation; Xist, X inactive specific transcript. Click here for file

Additional file 12 **Typical H3K27me3 patterns in XX ESCs upon differentiation.** Undifferentiated cells typically (shown are central sections) display a prominent enrichment of H3K27me3 at chromocenters (left panel). During an intermediate state H3K27me3-marked chromatin is distributed throughout the nucleus with slight enhancement around the nucleoli (middle panel), while distinct accumulation of H3K27me3 at the Barr body and complete exclusion from chromocenters is typically found around day 7 to 9 of differentiation (right panel). Scale bar: 5 μm. ESC, embryonic stem cell; H3K27me3, trimethylated histone H3 lysine 27. Click here for file

Additional file 13 **tr36 ESCs: Xist RNA in relation to painted CTs 11, karyotype analysis and variable Xist RNA expansion. (A)** Partial 3D reconstruction of two DAPI-stained undifferentiated tr36 ESC nuclei (red) and Xist RNA signals demonstrate the widespread distribution of Xist RNA into areas of decondensed chromatin. Scale bar: 5 μm. **(B)** Xist RNA (green) and painted CTs 11 (red) on a tr36 ESC nucleus demonstrate a radial Xist RNA spreading distinctly beyond the boundaries of a painted CT 11. Inset shows the whole nucleus including DAPI staining. Scale bar: 5 μm. **(C)** M-FISH karyotype analysis of the tr36 ESC line and respective frequencies of observed translocations involving chromosome 11. Circles indicate chromosomes 11. CT, chromosome territory; DAPI, 4',6-diamidino-2-phenylindole; ESC, embryonic stem cell; FISH, fluorescence *in situ* hybridization; Xist, X inactive specific transcript. Click here for file

Additional file 14 **Evidence for DNA replication within the ANC of Xi.** 3D-SIM mid z-sections of C2C12 cells fixed at different time points after a 5 min EdU pulse during replication of the Xi and PCNA immunodetection. The chase time between pulse labeling and fixation of each cell is indicated by the white bar in the time line. Upper insets show EdU and PCNA signals in Xi, lower insets show the respective chromatin environment with outlined positions of EdU and PCNA signals. **(A)** Fixation immediately after EdU pulse labeling reveals colocalization of nascent DNA and PCNA signals in decondensed regions of the ANC. **(B)** A similar picture is observed in cells with 10 min chase between EdU pulse and fixation. **(C)** A clear separation of EdU and PCNA signals becomes evident after a 20 min chase and **(D)** more pronounced after 60 min chase time. At these late time points, PCNA signals, marking the position of active replication forks are mostly observed at low DAPI intensity sites, while the EdU-marked DNA is repacked into more compacted chromatin. Scale bar: 2 μm, insets 0.5 μm. ANC, active nuclear compartment; 3D-SIM, three-dimensional structured illumination microscopy; DAPI, 4',6-diamidino-2-phenylindole; EdU, 5-ethynyl-2-deoxyuridine; PCNA, proliferating cell nuclear antigen; Xi, inactive X chromosome [48,121]. Click here for file

Additional file 15 **Workflow for quantitative analysis of 3D-SIM data.** Representative image of a C2C12 cell nuclei co-stained for Xist RNA (green) and a representative second nuclear marker (mSAF-A, red), counterstained with DAPI (grey). Evaluation steps performed with the TANGO plugin for ImageJ/Fiji [116] are highlighted with light blue, evaluation steps performed with a custom R-script are highlighted yellow. Insets in the middle column show input 3D-SIM image (left) and the respective result after segmentation/processing (right). Details are described in the Methods section. 3D-SIM, three-dimensional structured illumination microscopy; DAPI, 4',6-diamidino-2-phenylindole; SAF-A, scaffold attachment factor-A; Xist, X inactive specific transcript. Click here for file
