# Supplementary figures and images for: Three-dimensional super-resolution microscopy of the inactive X chromosome territory reveals a collapse of its active nuclear compartment harboring distinct Xist RNA foci
Source: Epigenetics Chromatin. 2014 Apr 28;7:8. doi: 10.1186/1756-8935-7-8 (PMC4108088; doi:10.1186/1756-8935-7-8)

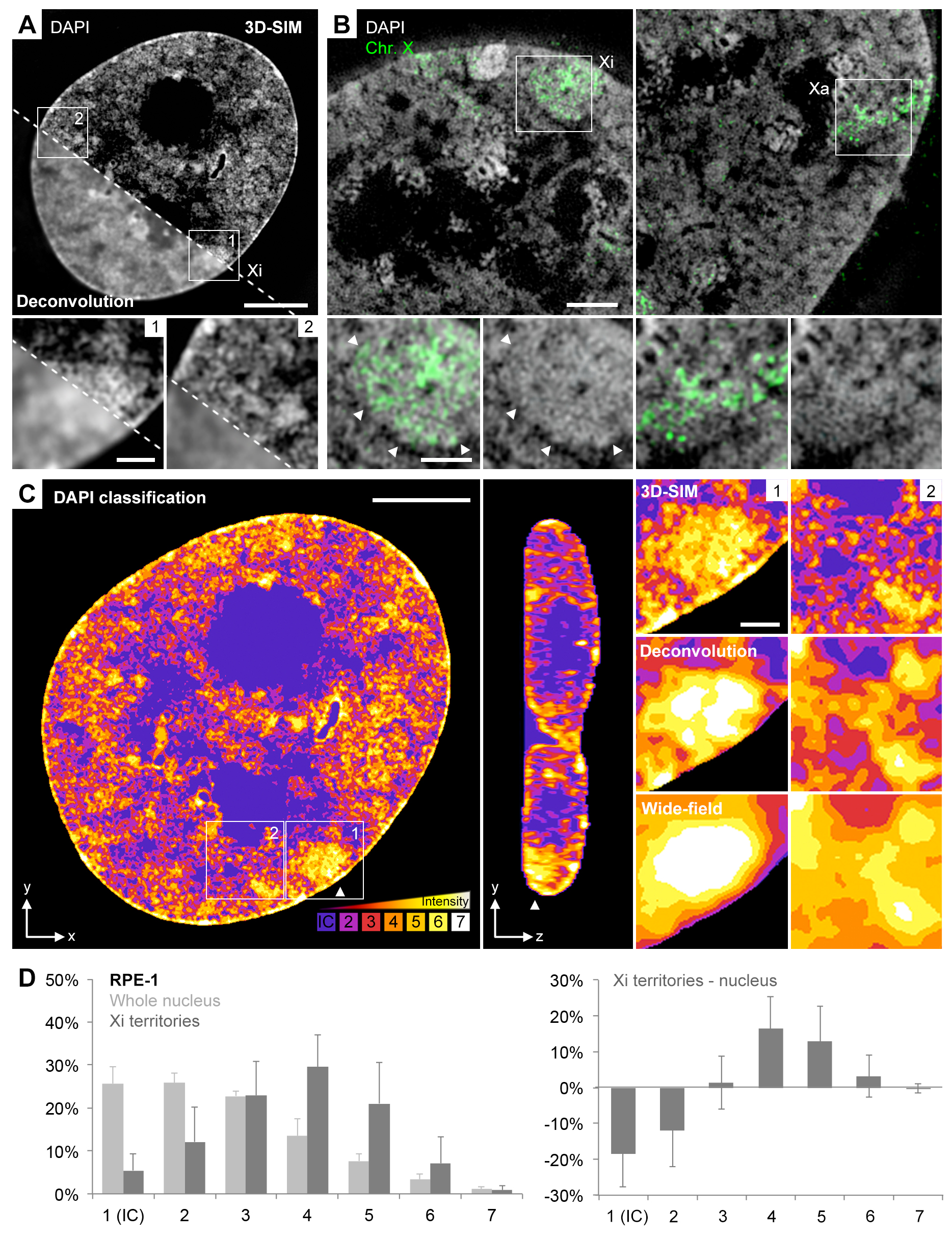

Supplement: Additional file 1 — 3D-SIM-based DAPI intensity classification in the Barr body versus the entire nucleus of RPE-1 cells. (A) Mid z-section of a DAPI-stained nucleus. The area below the dashed line illustrates the resolution level obtained by wide-field deconvolution microscopy, for comparison. Inset magnifications of the framed areas show the non-uniformly compacted structure of the Barr body resolvable with 3D-SIM (1) and an arbitrary autosomal region with CDCs (2). Scale bars: 5 μm, insets 1 μm. (B) X chromosome-specific painting (green) of Xi (left) and Xa territories (right) of the same nucleus in different z-sections. .Scale bars: 2 μm, insets 1 μm. (C) 3D DAPI intensity classification exemplified for the nucleus shown in (A). Seven DAPI intensity classes are displayed in false-color code ranging from class 1 (blue) representing pixels close to background intensity, largely representing the IC, up to class 7 (white) representing pixels with the highest density. Framed areas of the Barr body region (inset 1) and a representative autosomal region (inset 2) are shown as magnified on the right at the resolution levels of 3D-SIM, deconvolution and conventional wide-field microscopy. The non-uniformity of the Xi territory pervaded by areas of lower DAPI intensity classes becomes evident at 3D-SIM resolution, whereas both wide-field and deconvolution microscopy imply a concentric increase of density. In the representative autosomal region chromatin assigned to classes 2 to 3 lines compacted CDCs, represented by classes 4 to 6. (D) Left panel: average DAPI intensity classification profiles and respective standard deviations obtained from evaluation of entire nuclear volumes (n = 30 nuclei) or the Barr body region only (dark grey bars, n = 26 Barr bodies). Right panel: over/underrepresentation of the average DAPI intensity class fraction sizes in the Barr body versus entire nuclear volumes. 3D-SIM, three-dimensional structured illumination microscopy; CDC, chromatin domain cluster; DAPI, [file 1756-8935-7-8-S1.jpeg]

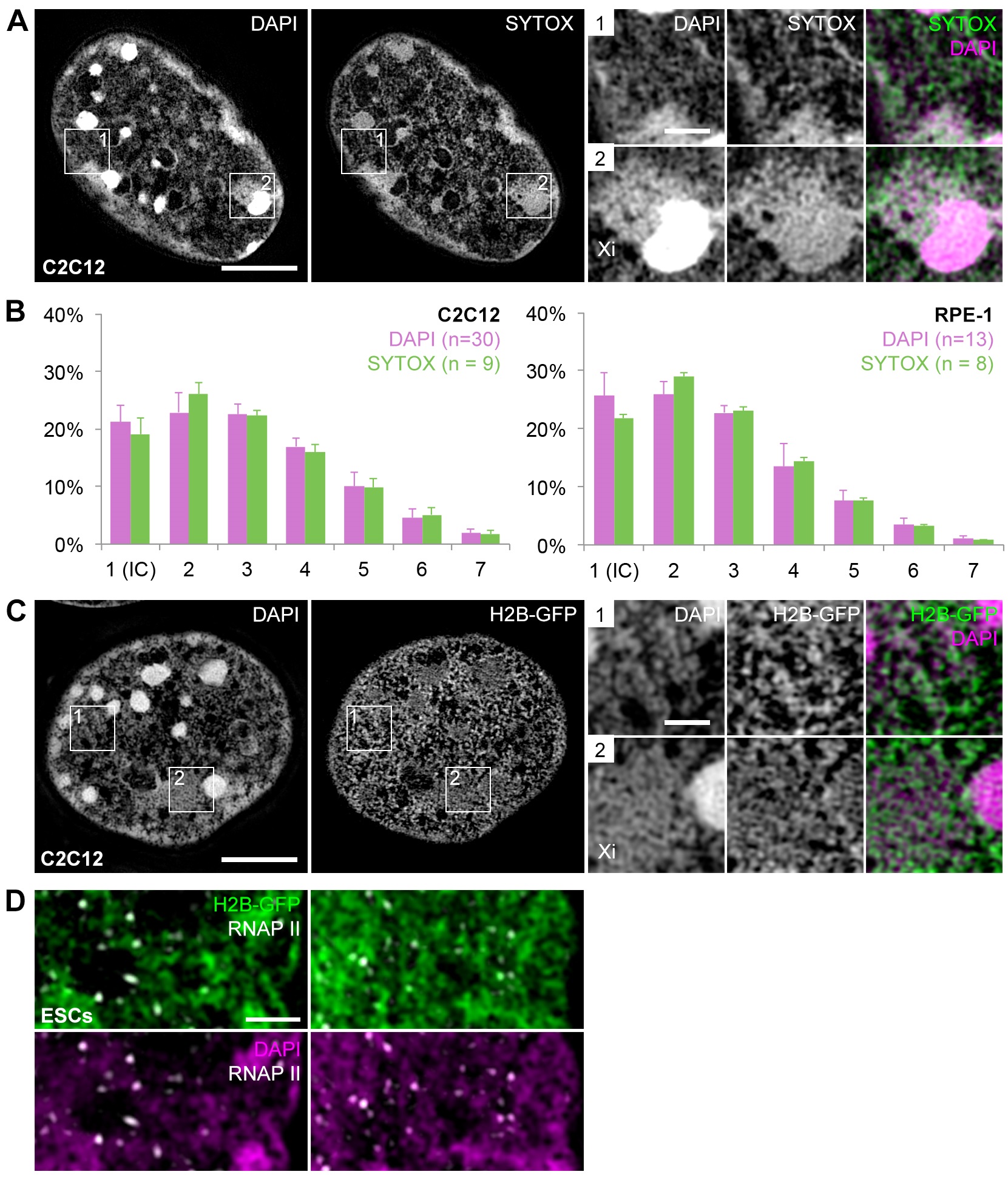

Supplement: Additional file 2 — Comparison of SYTOX, H2B-GFP and DAPI staining pattern by 3D-SIM. (A) Optical section of a DAPI- and SYTOX-stained C2C12 nucleus confirms the similar organization of compact CDCs, a high congruency of domain surfaces even at sites of decondensed chromatin and an IC channel system with a significant narrowing of the channels in the Barr body both after SYTOX and DAPI staining. The distinctly higher intensity of chromocenters in DAPI stains reflects their particularly high AT content. Inset magnifications show arbitrary autosomal regions (1) and the Barr body region (2). Scale bars: 5 μm, insets 1 μm. (B) Comparing histograms of DAPI and SYTOX intensity classification profiles for C2C12 and RPE-1 whole nuclei demonstrate similar intensity class distributions. (C) Optical section of a histone H2B-GFP expressed in a C2C12 cell nucleus shows, similar to DAPI, an organization of compact CDCs and an IC channel compartment that is narrowed in the Xi territory. Inset magnifications show an arbitrary autosomal region (inset 1) and the Xi territory (inset 2) as identified by H3K27me3 immunolabeling (not shown). Differences in the conformity of domain surfaces may in part be due to (cell cycle-dependent) uptake kinetics of transient H2B-GFP transfection. (D) Comparison of H2B-GFP stably expressed in an ESC cell line (green) and DAPI (magenta) staining together with RNAP II (grey) demonstrate a high conformity both at sites of decondensed and condensed chromatin. Scale bar: 1 μm. 3D-SIM, three-dimensional structured illumination microscopy; CDC, chromatin domain cluster; DAPI, 4',6-diamidino-2-phenylindole; ESC, embryonic stem cell; GFP, green fluorescent protein; H3K27me3, trimethylated histone H3 lysine 27; IC, interchromatin compartment; RNAP II, RNA polymerase II [52,117-120]. [file 1756-8935-7-8-S2.jpeg]

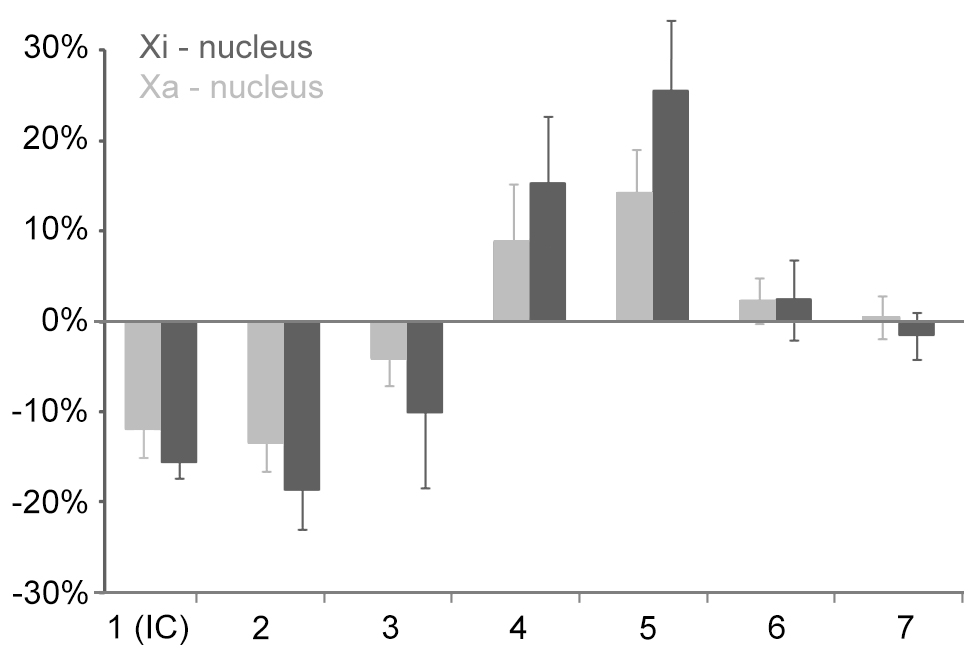

Supplement: Additional file 3 — DAPI intensity classification of painted Xa and Xi territories after 3D-FISH. The average AT content is approximately 59% in the human and 58% in the mouse genome with individual chromosomes ranging from approximately 52 to 63% in humans and 56 to 61% in mouse. With an AT content of approximately 61% in both human and mouse the X chromosome represents (the most) AT-rich chromosome [53]. It might thus be expected that X territories bind DAPI above average and therefore appear to have an increased compaction also on Xa. We therefore painted X territories in C2C12 cells by 3D-FISH and assessed DAPI intensity classification underneath the painted territories (as shown in Figure 1B). Density distributions within Xa territories were in fact intermediate between the entire nucleus and the Xi in line with the relatively high AT content of the X chromosome and possibly also with its overall low gene density and resulting low transcriptional activity (http://www.ncbi.nlm.nih.gov/mapview). The hybridized probe likely contributes in addition towards higher DAPI intensities underneath painted territories since DAPI fluorescence increases significantly when it is bound to double-stranded DNA [52], while the remaining nucleus may still harbor a significant fraction of single stranded DNA after denaturation. The histogram displays the under/overrepresentation of DAPI intensity classes relative to the entire nuclear chromatin region in painted Xa (light grey) and Xi territories (dark grey). Error bars mark the standard deviations. P <0.001 (n = 5 cells). DAPI, 4',6-diamidino-2-phenylindole; FISH, fluorescence in situ hybridization; Xa, active X chromosome; Xi, inactive X chromosome. [file 1756-8935-7-8-S3.jpeg]

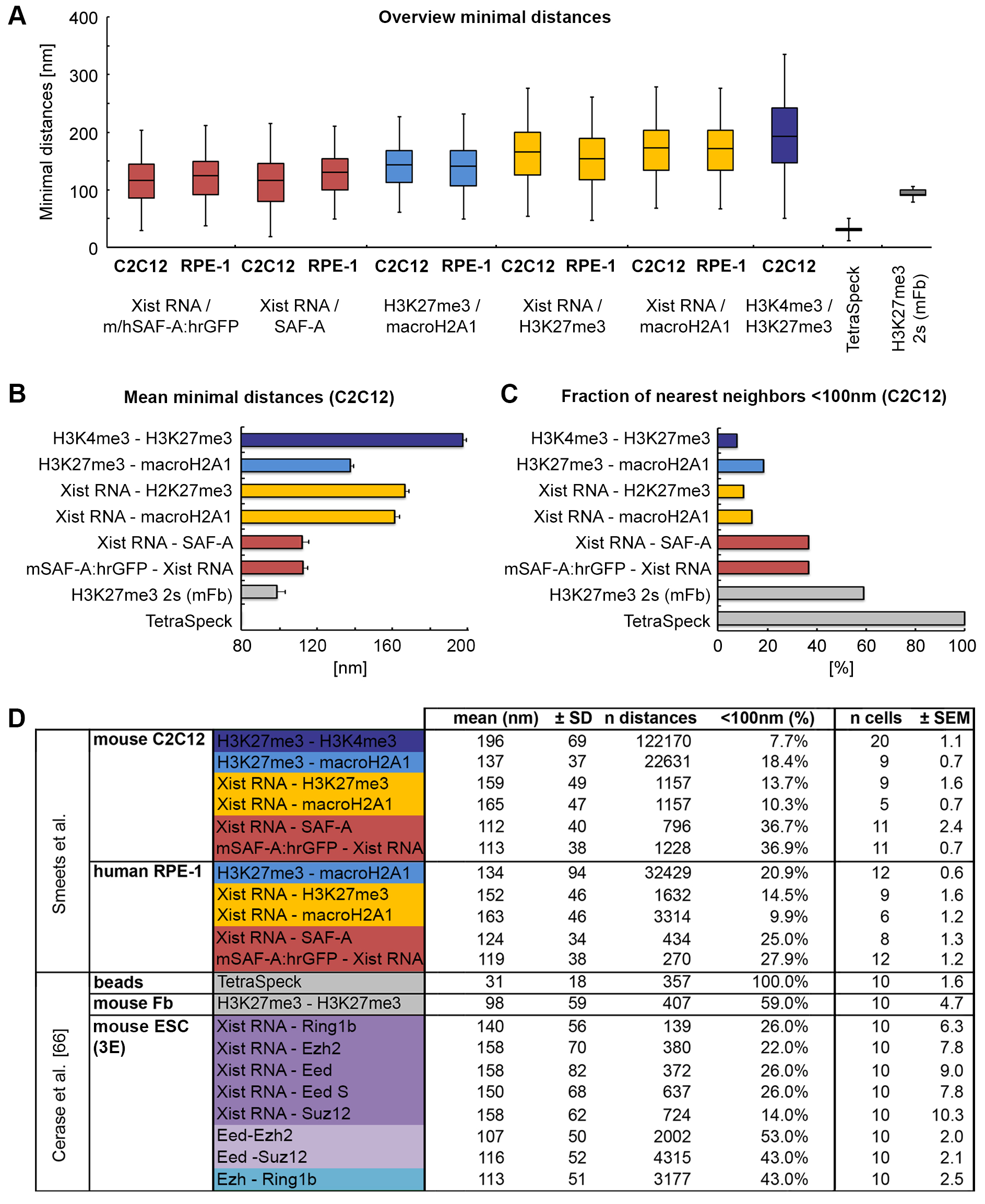

Supplement: Additional file 4 — Overview of nearest neighbor measurements. Distance measurements between histone markers (indicated by blue bars), between Xist RNA and histone markers (yellow bars), Xist RNA and SAF-A (red bars) were performed in this study. Control measurements (grey bars) were taken from a parallel study [66]. (A) Minimal distance distributions combined from all cells with their medians displayed as box plots (median, Q1, Q3) with whiskers indicating the 1.5 IQR. (B) Mean of the mean values obtained for individual cells with their respective error (SEM) are displayed demonstrating a very small variation between individual cell measurements. (C) Fraction of nearest neighbor distances below 100 nm. (D) List of all distance measurements performed in this study and in a parallel study [66]. As ‘colocalization’ control mouse fibroblasts were labeled with primary antibodies against H3K27me3, which were simultaneously detected with two secondary antibody species conjugated to different dyes. Their average minimal distance was approximately 100 nm with 60% of minimal distances below 100 nm, which reflects the collective ‘error’ of the applied method due to detection/labeling specificity, optical mismatch and evaluation inaccuracies (discussed in detail in Cerase et al. [66]). 1.5 IQR, 1.5 × interquartile range; H3K27me3, trimethylated histone H3 lysine 27; SAF-A, scaffold attachment factor-A; SEM, standard error of the mean; Xist, X inactive specific transcript. [file 1756-8935-7-8-S4.jpeg]

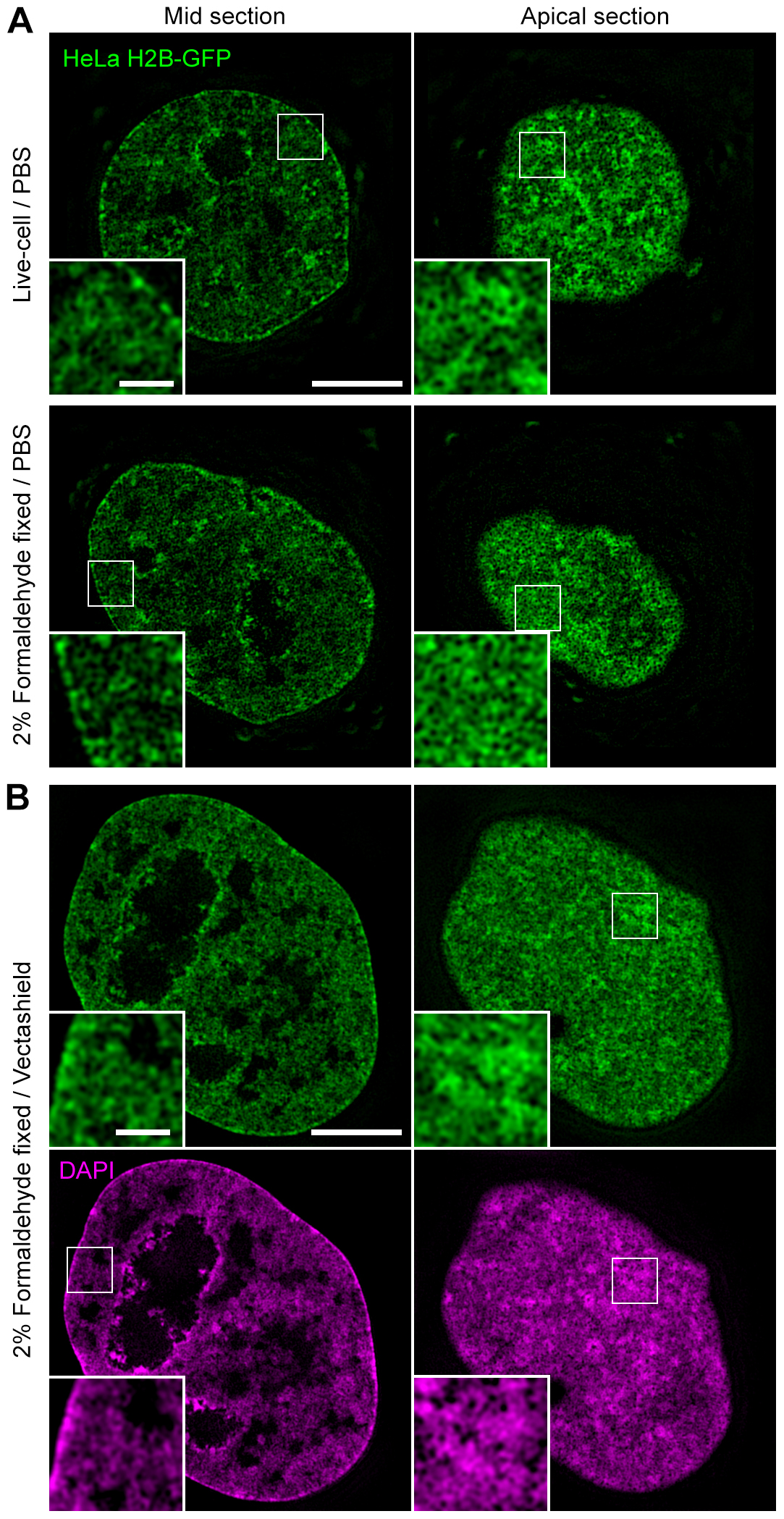

Supplement: Additional file 8 — Super-resolution imaging of histone H2B-GFP in living and fixed cells. (A) HeLa cells stably expressing histone H2B-GFP imaged in the living state (upper panel) and after formaldehyde fixation (lower panel). Basic features of chromatin organization, for example compact CDCs, sites of decondensed chromatin, IC (lacunae) leading to nuclear pores, are similar between living and fixed cells. Scale bar: 5 μm, insets 1 μm. (B) Comparison of H2B-GFP (green) and DAPI (magenta) staining to evaluate DAPI’s overall chromatin coverage capacity and its general suitability as a marker of chromatin after fixation in formaldehyde, permeabilization and mounting in Vectashield. DAPI and H2B-GFP show a high conformity even at sites of decondensed chromatin or at chromatin voids at nuclear pores. Scale bar: 5 μm, insets 1 μm. CDC, chromatin domain cluster; DAPI, 4',6-diamidino-2-phenylindole; GFP, green fluorescent protein; IC, interchromatin compartment. [file 1756-8935-7-8-S8.jpeg]

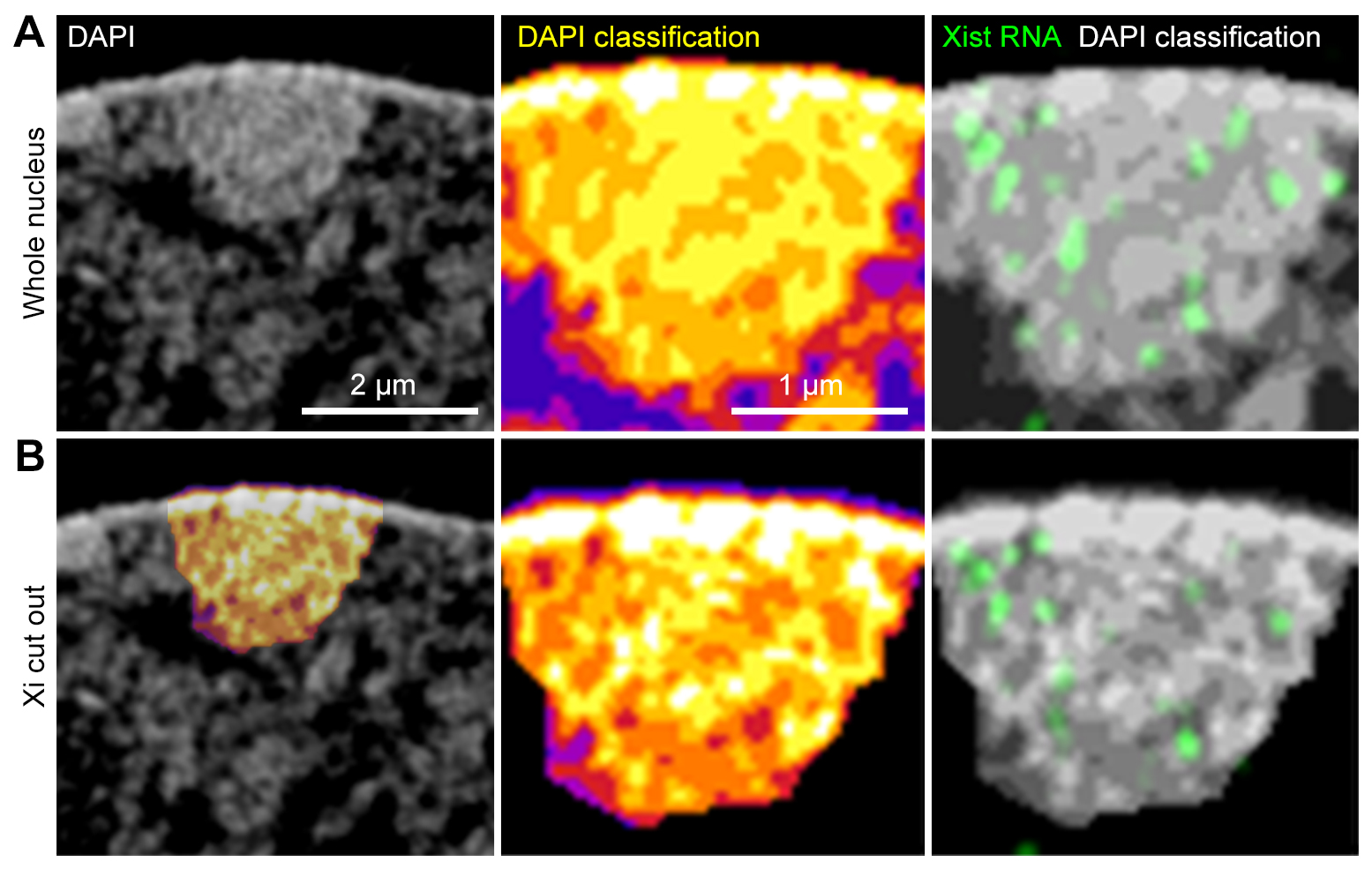

Supplement: Additional file 9 — Detailed illustration of Xist RNA localization within low DAPI intensity sites. (A) DAPI intensity profile in a mid z-section through the Barr body of a DAPI-stained C2C12 nucleus after classification of the entire nucleus. Right panel shows Xist RNA foci (green) in the respective section after conversion of classified DAPI intensities into greyscale. (B) Same nucleus after classification only of the Barr body unravels a broader stretching and more detailed chromatin classification. Overlay with the Xist RNA signal depicts the preferential localization of Xist RNA within low chromatin density classes (right). DAPI, 4',6-diamidino-2-phenylindole; Xist, X inactive specific transcript. [file 1756-8935-7-8-S9.jpeg]

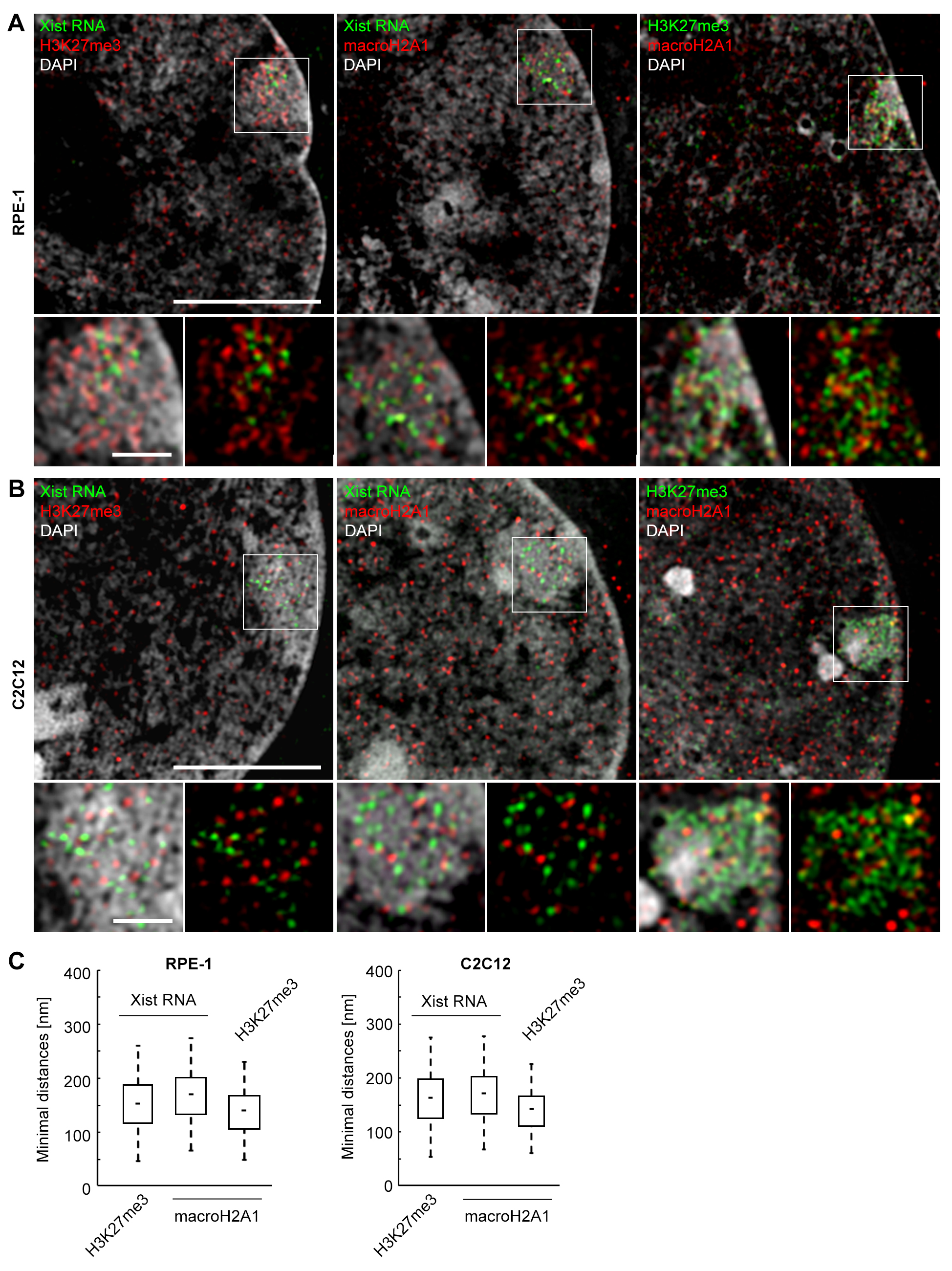

Supplement: Additional file 10 — Spatial association of Xist RNA with H3K27me3- and macroH2A1-marked chromatin in RPE-1 and C2C12 nuclei. Mid z-sections of DAPI-stained (A) RPE-1 and (B) C2C12 nuclei after immuno-RNA-FISH against Xist RNA (green) and H3K27me3 (red), Xist RNA and macroH2A1 (red), or immunodetection of H3K27me3 (green) and macroH2A1 (red). Inset magnifications of depicted areas demonstrate the distinct localization of Xist RNA from both H3K27me3 and macroH2A1 and a partial overlap between H3K27me3 and macroH2A1. Scale bar: 5 μm, insets 1 μm. (C) Boxplots of minimal distance distributions between Xist RNA signals and H3K27me3 or mH2A1 as well as between H3K27me3 or mH2A1. DAPI, 4',6-diamidino-2-phenylindole; FISH, fluorescence in situ hybridization; H3K27me3, trimethylated histone H3 lysine 27; Xist, X inactive specific transcript. [file 1756-8935-7-8-S10.jpeg]

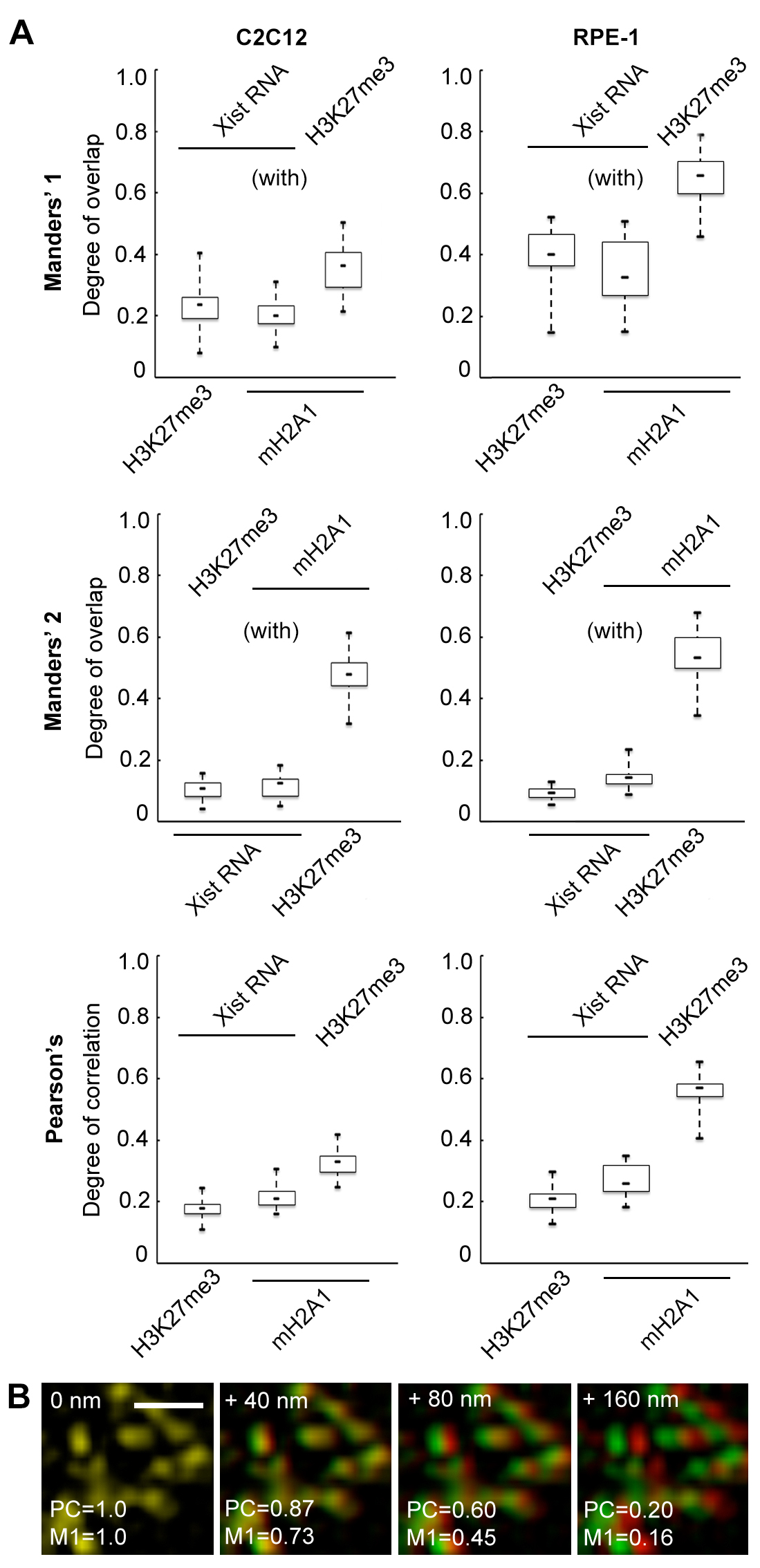

Supplement: Additional file 11 — Colocalization analysis using Pearson’s and Manders’ correlation coefficients. (A) Quantification of colocalization between H3K27me3 and macroH2A1 with Xist RNA in C2C12 (left column) and RPE-1 cells (right column) using Manders’ (M1 and M2) and Pearson’s correlation (PC) coefficients (n = 10 nuclei per evaluation). (B) Colocalization coefficients become more stringent with increasing optical resolution. To link visual impression, numerical colocalization values and absolute distances obtained by 3D-SIM, different degrees of (partial) colocalization were simulated in an idealized example. One single greyscale image showing objects in the size range of approximately 100 to 200 nm was copied into two color channels (green and red, distance = 0 nm; complete overlap = yellow; Pearson’s coefficient (PC) = 1.0; Manders’ coefficient (M1) = 1.0). Subsequent shifts of the red against the green channel at indicated lengths in x-direction reveal visual separation of the differently colored objects at distances of >40 nm. A first clear separation of the two channels is seen at distances of approximately 80 nm, while shifting of 160 nm shows a clear gap between the signals of the two channels. PC as well as M1 coefficients are indicated at each step. Scale bar: 0.5 μm. 3D-SIM, three-dimensional structured illumination microscopy; H3K27me3, trimethylated histone H3 lysine 27; PC, Pearson’s correlation; Xist, X inactive specific transcript. [file 1756-8935-7-8-S11.jpeg]

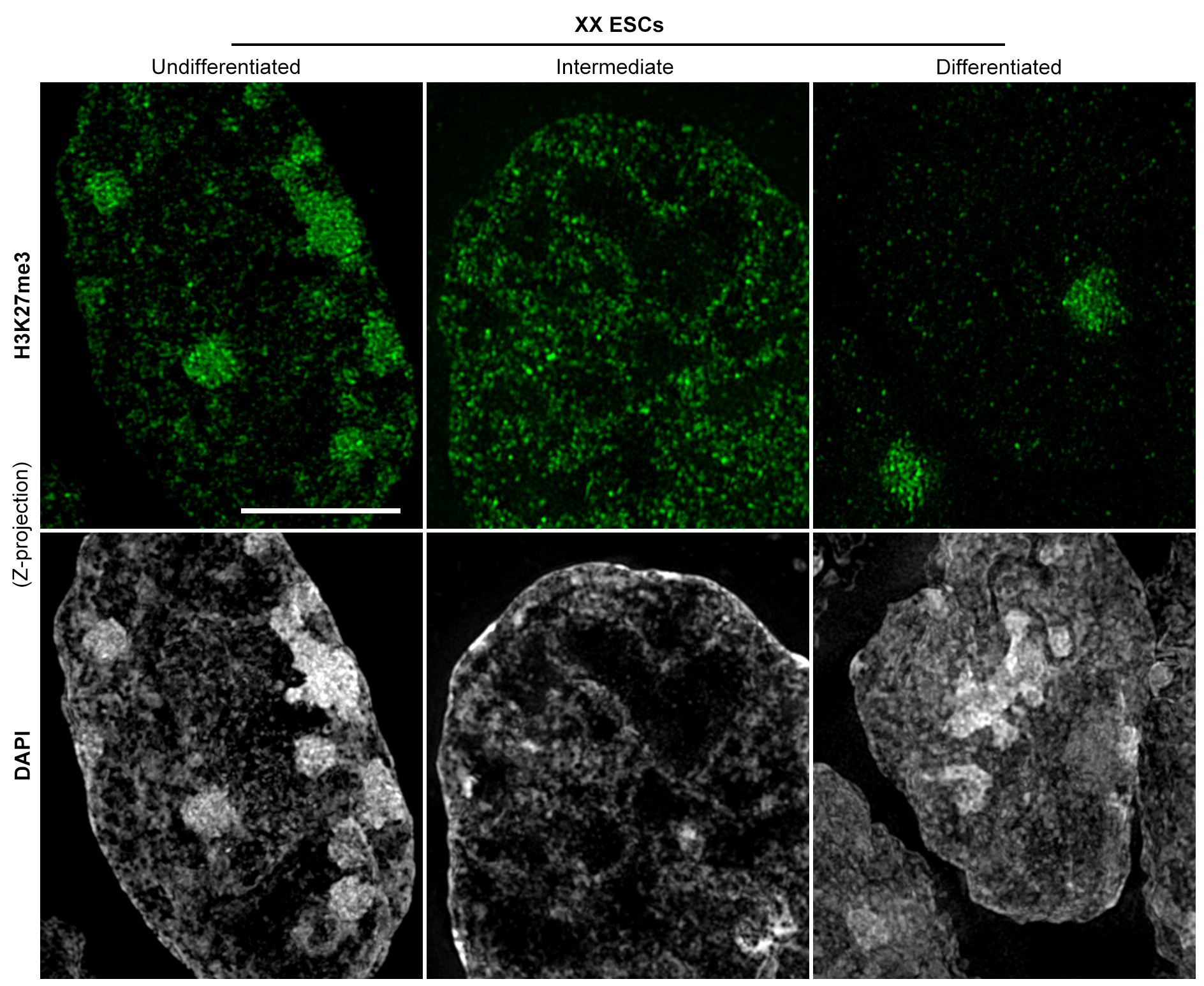

Supplement: Additional file 12 — Typical H3K27me3 patterns in XX ESCs upon differentiation. Undifferentiated cells typically (shown are central sections) display a prominent enrichment of H3K27me3 at chromocenters (left panel). During an intermediate state H3K27me3-marked chromatin is distributed throughout the nucleus with slight enhancement around the nucleoli (middle panel), while distinct accumulation of H3K27me3 at the Barr body and complete exclusion from chromocenters is typically found around day 7 to 9 of differentiation (right panel). Scale bar: 5 μm. ESC, embryonic stem cell; H3K27me3, trimethylated histone H3 lysine 27. [file 1756-8935-7-8-S12.jpeg]

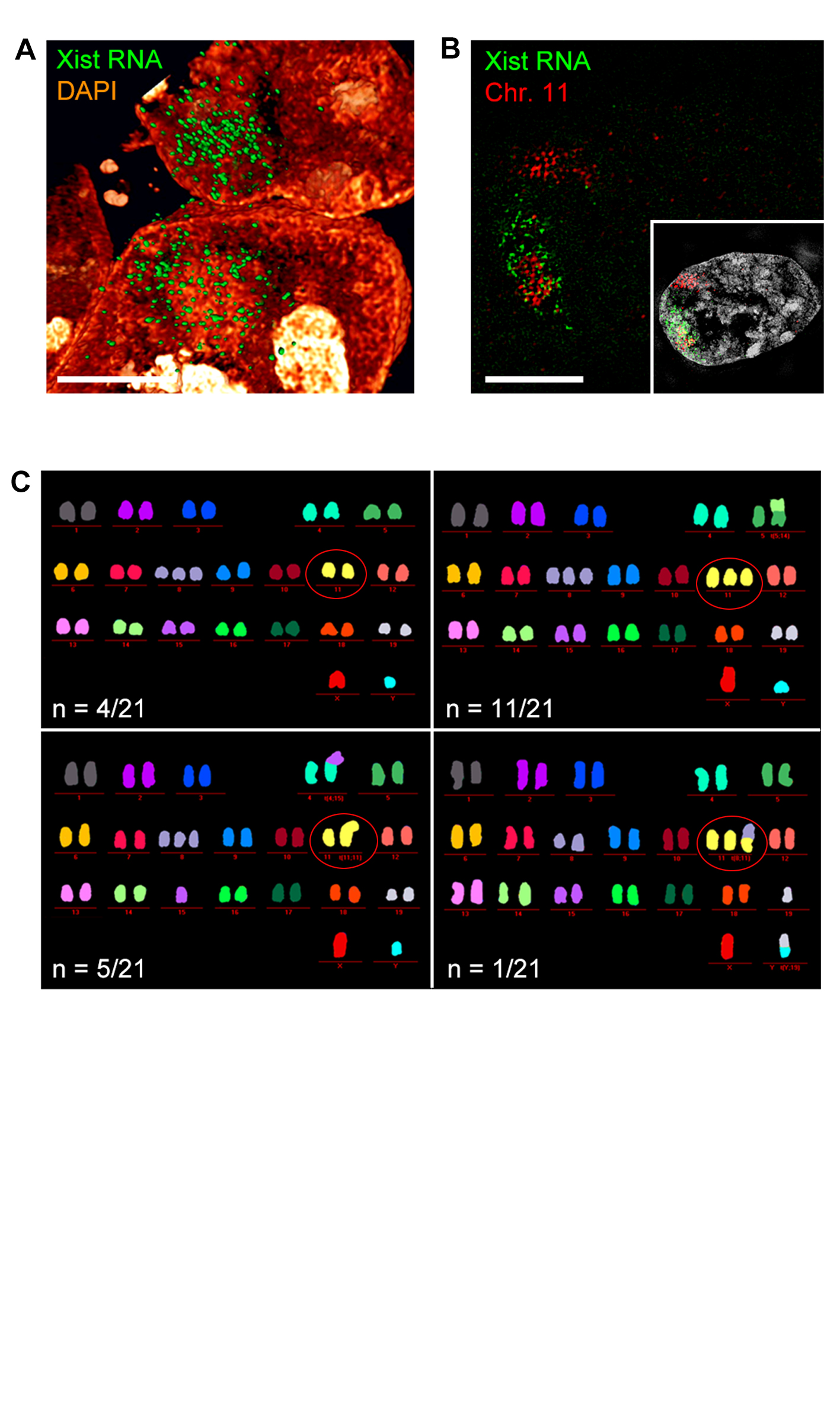

Supplement: Additional file 13 — tr36 ESCs: Xist RNA in relation to painted CTs 11, karyotype analysis and variable Xist RNA expansion. (A) Partial 3D reconstruction of two DAPI-stained undifferentiated tr36 ESC nuclei (red) and Xist RNA signals demonstrate the widespread distribution of Xist RNA into areas of decondensed chromatin. Scale bar: 5 μm. (B) Xist RNA (green) and painted CTs 11 (red) on a tr36 ESC nucleus demonstrate a radial Xist RNA spreading distinctly beyond the boundaries of a painted CT 11. Inset shows the whole nucleus including DAPI staining. Scale bar: 5 μm. (C) M-FISH karyotype analysis of the tr36 ESC line and respective frequencies of observed translocations involving chromosome 11. Circles indicate chromosomes 11. CT, chromosome territory; DAPI, 4',6-diamidino-2-phenylindole; ESC, embryonic stem cell; FISH, fluorescence in situ hybridization; Xist, X inactive specific transcript. [file 1756-8935-7-8-S13.jpeg]

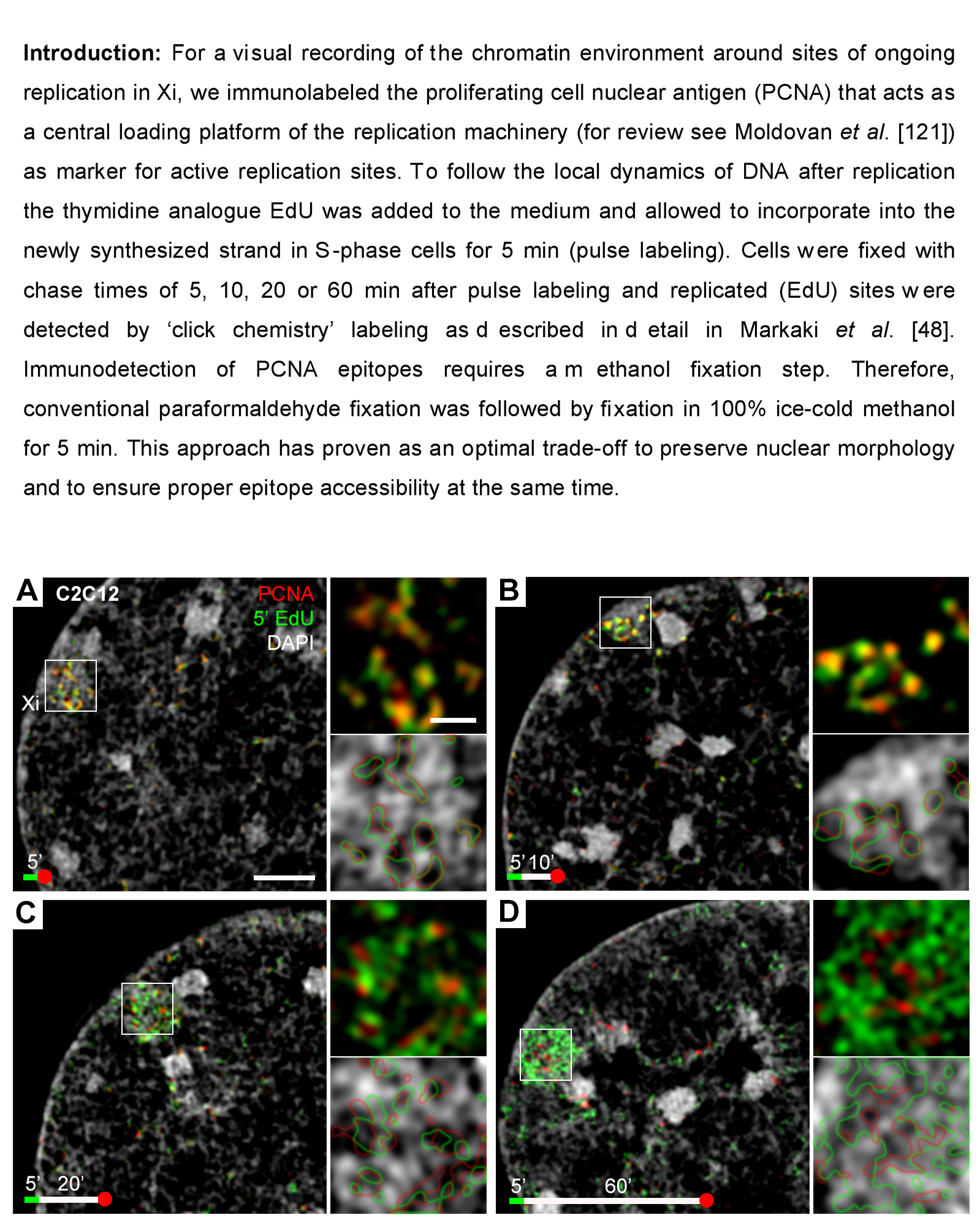

Supplement: Additional file 14 — Evidence for DNA replication within the ANC of Xi. 3D-SIM mid z-sections of C2C12 cells fixed at different time points after a 5 min EdU pulse during replication of the Xi and PCNA immunodetection. The chase time between pulse labeling and fixation of each cell is indicated by the white bar in the time line. Upper insets show EdU and PCNA signals in Xi, lower insets show the respective chromatin environment with outlined positions of EdU and PCNA signals. (A) Fixation immediately after EdU pulse labeling reveals colocalization of nascent DNA and PCNA signals in decondensed regions of the ANC. (B) A similar picture is observed in cells with 10 min chase between EdU pulse and fixation. (C) A clear separation of EdU and PCNA signals becomes evident after a 20 min chase and (D) more pronounced after 60 min chase time. At these late time points, PCNA signals, marking the position of active replication forks are mostly observed at low DAPI intensity sites, while the EdU-marked DNA is repacked into more compacted chromatin. Scale bar: 2 μm, insets 0.5 μm. ANC, active nuclear compartment; 3D-SIM, three-dimensional structured illumination microscopy; DAPI, 4',6-diamidino-2-phenylindole; EdU, 5-ethynyl-2-deoxyuridine; PCNA, proliferating cell nuclear antigen; Xi, inactive X chromosome [48,121]. [file 1756-8935-7-8-S14.doc]

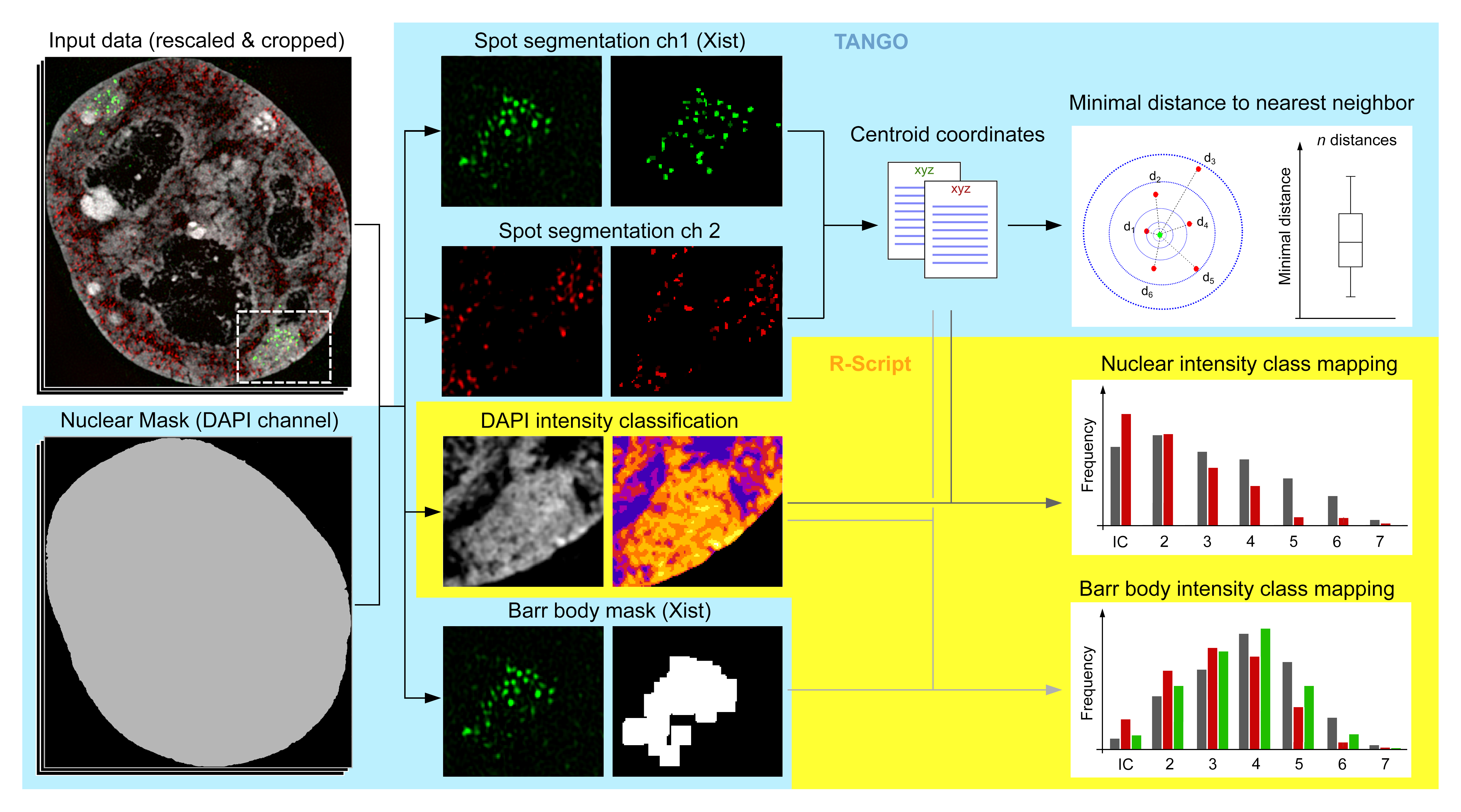

Supplement: Additional file 15 — Workflow for quantitative analysis of 3D-SIM data. Representative image of a C2C12 cell nuclei co-stained for Xist RNA (green) and a representative second nuclear marker (mSAF-A, red), counterstained with DAPI (grey). Evaluation steps performed with the TANGO plugin for ImageJ/Fiji [116] are highlighted with light blue, evaluation steps performed with a custom R-script are highlighted yellow. Insets in the middle column show input 3D-SIM image (left) and the respective result after segmentation/processing (right). Details are described in the Methods section. 3D-SIM, three-dimensional structured illumination microscopy; DAPI, 4',6-diamidino-2-phenylindole; SAF-A, scaffold attachment factor-A; Xist, X inactive specific transcript. [file 1756-8935-7-8-S15.jpeg]
